# Prognostic significance of PLIN1 expression in human breast cancer

**DOI:** 10.18632/oncotarget.10239

**Published:** 2016-06-23

**Authors:** Cefan Zhou, Ming Wang, Li Zhou, Yi Zhang, Weiyong Liu, Wenying Qin, Rong He, Yang Lu, Yefu Wang, Xing-Zhen Chen, Jingfeng Tang

**Affiliations:** ^1^ Institute of Biomedical and Pharmaceutical Sciences, and Provincial Cooperative Innovation Center, College of Bioengineering, Hubei University of Technology, Wuhan, Hubei, China; ^2^ The State Key Laboratory of Virology, College of Life Sciences, Wuhan University, Wuhan, Hubei, China; ^3^ Department of Clinical Laboratory, Renmin Hospital of Wuhan University, Wuhan, Hubei, China; ^4^ Animal Biosafety Level III Laboratory at the Center for Animal Experiment, Wuhan University, Wuhan, China; ^5^ Department of Clinical Laboratory, Tongji Hospital, Tongji Medical College of Huazhong University of Science and Technology, Wuhan, Hubei, China; ^6^ Membrane Protein Disease Research Group, Department of Physiology, Faculty of Medicine and Dentistry, University of Alberta, Edmonton, AB, Canada

**Keywords:** perilipin-1, Kaplan-Meier analysis, meta analysis, tumor suppressor, biomarker

## Abstract

Breast cancer is a heterogeneous disease associated with diverse clinical, biological and molecular features, presenting huge challenges for prognosis and treatment. Here we found that perilipin-1 (PLIN1) mRNA expression is significantly downregulated in human breast cancer. Kaplan-Meier analysis indicated that patients presenting with reduced PLIN1 expression exhibited poorer overall metastatic relapse-free survival (*p* = 0.03). Further Cox proportional hazard models analysis revealed that the reduced expression of PLIN1 is an independent predictor of overall survival in estrogen receptor positive (*p* < 0.0001, HR = 0.87, 95% CI = 0.81–0.92, *N* = 3,600) and luminal A-subtype (*p* = 0.02, HR = 0.88, 95% CI = 0.78–0.98, *N* = 1,469) breast cancer patients. We also demonstrated that the exogenous expression of PLIN1 in human breast cancer MCF-7 and MDA-MB-231 cells significantly inhibits cell proliferation, migration, invasion and *in vivo* tumorigenesis in mice. Together, these data provide novel insights into a prognostic significance of PLIN1 in human breast cancer and reveal a potentially new gene therapy target for breast cancer.

## INTRODUCTION

Breast cancer is the most common malignancy among women (i.e., 200,000 new cases diagnosed each year in the United States) and represents an important worldwide public health issue [1, 2]. It is a complex disease that is caused by multiple genetic and environmental factors and is recognized as a major cause of cancer-related death in women. The treatment of breast cancer is particularly difficult in patients with metastatic tumors [3]. Although progress has been made in the diagnosis and treatment of breast cancer, the prognosis and survival for most patients, particularly those with metastases, have not dramatically improved [4, 5]. Therefore, there is an urgent need for the identification of diagnostic markers and potential cellular and molecular mechanisms underlying tumor metastasis, as well as for the development of new therapeutic strategies for improving patient survival and overall quality of life.

The application of next generation sequencing technologies to mRNA sequencing (RNA-Seq) is a widely used approach in transcriptomic studies [6, 7]. RNA-Seq provides information for expression analysis at the transcript level and overcomes the limitations of cross-hybridization and restricted ranges of the measured expression levels compared with microarray technologies [8]. The generations of publicly available large-scale datasets, such as the Cancer Genome Atlas (TCGA), provide comprehensive catalogs of multiple data types performed on the same set of samples. Various groups have identified large multi-gene signatures that are prognostic for outcomes in molecularly profiled human breast cancer samples through the TCGA database [9–11].

Here, we sought to identify single-gene prognostic biomarkers using a meta-analysis of publicly available mRNA expression data. We first analyzed the expression patterns of genes in breast cancer obtained from the Cancer Genome Atlas (TCGA) database and screened for dysregulated gene entities. Combining the results of gene ontology and protein interaction network analyses, we found PLIN1 exhibits significantly reduced expression in breast cancer samples compared with normal controls.

PLIN1 is a member of the PAT protein family that consists of adipose differentiation-related protein (ADRP), 47-kD tail-interacting protein (TIP47), S3-12 and OXPAT, and plays distinct roles in regulating both triglyceride storage and lipolysis in adipocytes. It has been regarded as a candidate gene that contributes to the highly complex, polygenic disease phenotype of human obesity [12]. Localized on the surface of intracellular lipid droplets, PLIN1 coordinates the access of other proteins (lipases) to the lipid esters within the lipid droplet core and can interact with cellular machinery that is important for lipid droplet biogenesis [13, 14]. Proteomic studies have determined that PLIN1 is also involved in intracellular trafficking, signaling, chaperone function, RNA metabolism and cytoskeletal organization [15–19]. Moreover, experimental studies using *in vitro* models have shown that triglyceride-rich remnant-like particles can induce carcinogenesis by upregulating the MEK/ERK and Akt pathways, which are involved in controlling cellular growth and proliferation, apoptosis, cell cycle arrest and lipid biosynthesis [20, 21]. Although alterations in lipid metabolism in cancer cells have received limited attention, their importance has become increasingly recognized [22–25]. However, the role of PLIN1 in human cancer, particularly in human breast cancer, remains unknown.

To determine whether PLIN1 is a potential prognostic biomarker for breast cancer, we assessed the mRNA levels of PLIN1 in human breast cancer tissues, as well as the role of PLIN1 in human breast cancers. We further investigated the correlation of PLIN1 mRNA levels with prognostic significance in human breast cancers by performing a meta-analysis using the Bc-GenExMiner v3.2 database. A receiver operating characteristic (ROC) curve was also generated to explore whether PLIN1 is a good diagnostic marker for discriminating tumor tissues from normal tissues.

## RESULTS

### Identification of biomarkers in breast cancer

**Figure 1:**
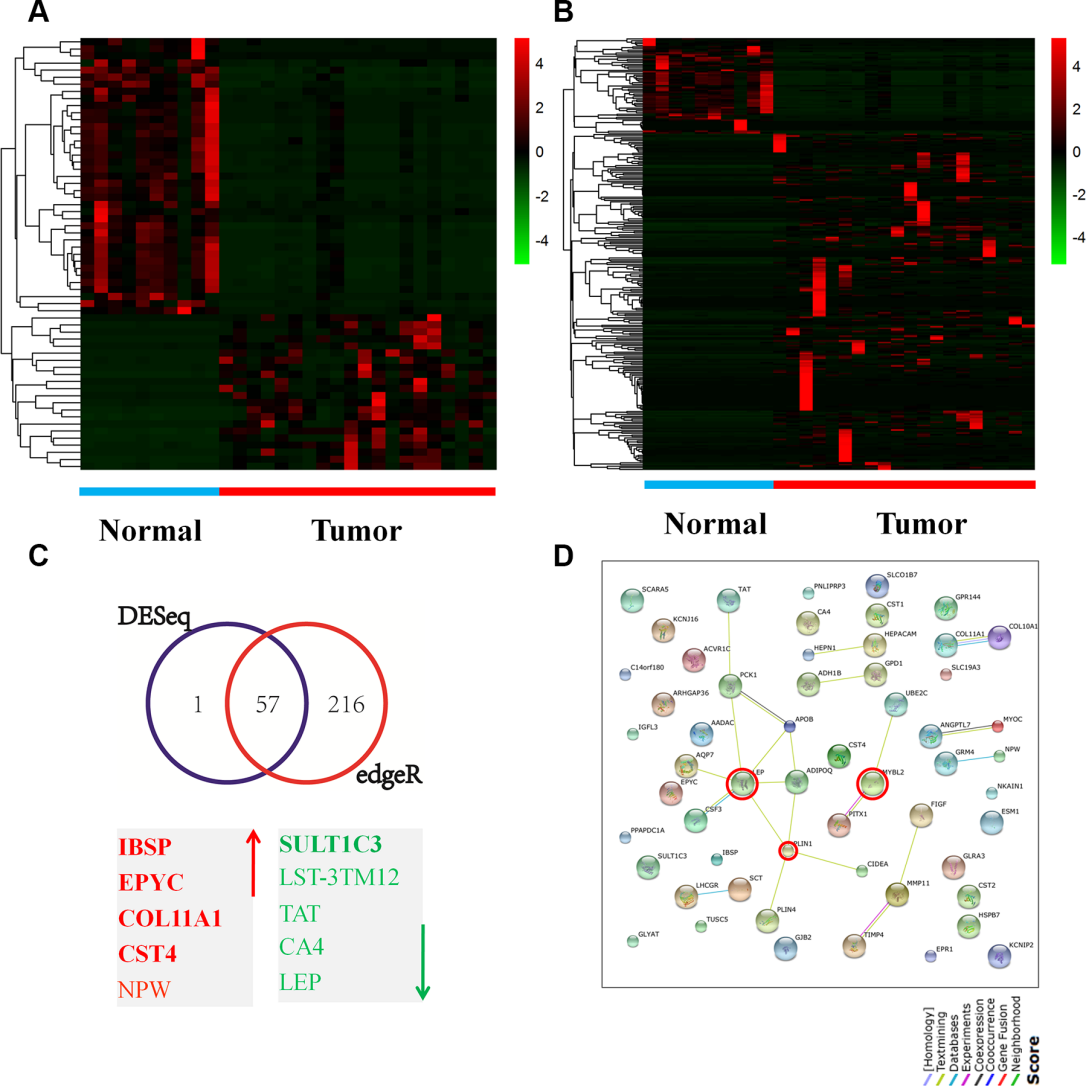
Gene expression patterns in breast cancer tissues (**A–B**) A heatmap illustrating genes expression profiles for the 30 breast cancer cases (10 normal and 20 tumors). The log2 values were calculated for each sample by normalizing to read counts alone (log2Fold Change). Heatmap analysis was performed by R version 3.2.2 software with DESeq package (*p* < 0.05 and log2 Fold Change > 4) (A) and edgeR package (*p* < 0.05 and log2 Fold Change > 4) (B). Short red and green vertical bars indicate upregulated and downregulated genes, respectively. RNAseq data were downloaded from TCGA database. (**C**) A Venn diagram of the concordant gene entities by the two algorithms and the top 10 genes, of which 5 were upregulated (red) and 5 were downregulated (green). (**D**) Analysis for protein-protein interaction by the STRING network identified two major interconnecting clusters with high-degree interactions between the genes (*N* = 57); three nodes of connection were encircled.

We first investigated the expression patterns of genes in breast cancer tissues. RNA-seq datasets for 208 tumor and 99 normal tissue samples were downloaded from The Cancer Genome Atlas (TCGA). Among these datasets, 30 (tumor = 20, normal = 10) were used to generate a heatmap for further analysis with the R program (Version 3.2.2) using “DESeq” and “edgeR” algorithms. A statistically significant gene list with a log2FC > 4.0 and a *p*-value of < 0.01 was used for further analysis. A total of 58 genes (21 upregulated and 37 downregulated) were identified by DESeq analysis (Figure 1A), whereas 276 genes (215 upregulated and 61 downregulated) were identified by edgeR analysis (Figure 1B). Microsoft Access analysis across these gene lists revealed a list of 57 concordant genes entities (21 upregulated and 36 downregulated) (Figure 1C and Supplementary Table 1). STRING database analyses identified a network of interactions between 57 of the genes mentioned above and showed three nodes of connection (Figure 1D).

The 57 concordant genes list was cross-compared with the cBioPortal database to assess the mutation, copy number and mRNA expression status in a human breast cancer cohort (*N* = 1,105). Twenty-nine genes were altered in at least 5% of the patients examined, whereas a subset of 5 genes were altered in at least 10% of the samples at the mutation, copy number and mRNA levels (Supplementary Figure 1A).

The significantly enriched Gene Ontology (GO) functional terms yielded by a DAVID ontology enrichment search (http://david.abcc.ncifcrf.gov/), which included biological processes, molecular functions and cellular components, are presented in Supplementary Figure 1. Predominant molecular functions included transport activity (21.4%; *N* = 18), channel activity (20.2%, *N* = 17) and binging process (19.0%, *N* = 16) (Supplementary Figure 1B). Among biological processes, biological regulation (32.2%, *N* = 73) exhibited maximal representation, with regulation of lipid metabolic processes as the predominant component (Supplementary Figure 1C). For cellular components, extracellular regions (58.3%, *N* = 35) were a predominant term (Supplementary Figure 1D). Three KEGG pathways were enriched within these signatures (Supplementary Figure 1E), which are predominantly involved in lipid metabolism.

To identify biomarkers with high prognostic significance in human breast cancers, 11 additional genes were selected from the 57 genes list based on the following triple criteria: genes with a log2FC value of more than 7.0, including integrin binding sialoprotein (IBSP), epiphycan (EPYC), collagen type XI alpha 1 (COL11A1), cystatin S (CST4), sulfotransferase family 1C member 3 (SULT1C3) and ubiquitin-conjugating enzyme E2C (UBE2C); nodes within the String database, including leptin (LEP), v-myb avian myeloblastosis viral oncogene homolog-like 2 (MYBL2) and perilipin 1 (PLIN1); and subsets of genes with alterations in at least 10% of patients (mutation, copy number and expression) within the cBioPortal database, which included carbonic anhydrase IV (CA4), myocilin, trabecular meshwork inducible glucocorticoid response (MYOC) and colony stimulating factor 3 (CSF3). Taken together, these exploratory analyses suggest an important functional role of lipid metabolic processes in the regulation of breast cancer progression.

### Expression levels of PLIN1 correlate with breast cancer patient survival

**Figure 2:**
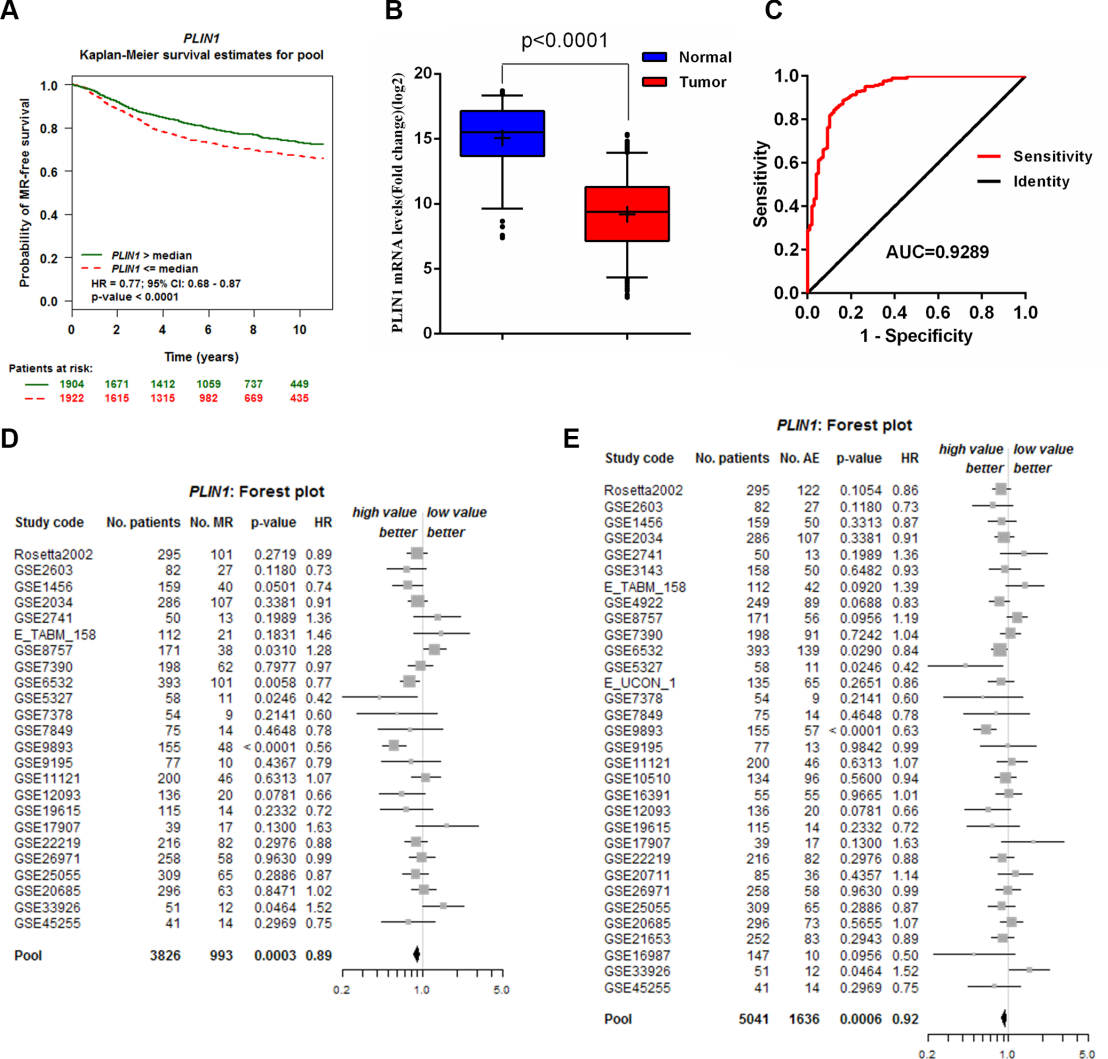
Validation of the PLIN1 gene signature for predicting survival (**A**) Kaplan-Meier MR-free overall survival curves using the Bc-GenExMiner v3.2 database (*N* = 3826). (**B**) PLIN1 mRNA expression was downregulated in 308 breast cancer samples downloaded from TCGA database. (**C**) ROC curve of PLIN1 expression in breast cancer patients from normal subjects. The area under the curve (AUC) was 0.93, with a standard error of 0.02 and a 95% confidence interval of 0.89–0.96. (**D–E**) Forest plots of PLIN1 expression on MR-free (D) and AE-free (E) survival.

Kaplan-Meier analysis was used to investigate whether the expression levels of the identified biomarkers correlated with the overall metastatic relapse-free (MR- free) survival of breast cancer patients using the Bc-GenExMiner v3.2 database [26]. Patients exhibiting abnormal expression of four genes, including PLIN1 (*p* = 0.03), IBSP (*p* = 0.0005), COL11A1 (*p* = 0.04), MYBL2 (*p* < 0.00001) and UBE2C (*p* < 0.00001) (Figure 2A, Supplementary Figure 2 and Supplementary Figure 3A), showed an adverse clinical outcome. The elevated expression of IBSP, COL11A1, MYBL2 and UBE2C in breast cancer has previously been reported to directly correlate with tumor progression [27–30]. Perilipins are the most abundant proteins at the surfaces of lipid droplets in adipocytes and PLIN1 plays a crucial role in the regulation of lipid metabolic processes [13]. However, the effects of PLIN1 in breast cancer remain unknown. The expression of PLIN1 was significantly downregulated in 307 breast cancer samples from the TCGA database (Figure 2B). ROC curves were generated and indicated that the PLIN1 mRNA levels in breast cancer samples differ significantly from those observed in control samples, with an AUC value of 0.93 (Figure 2C). Using the cutoff value of 9.68, the sensitivity and specificity values of 0.55 and 0.96, respectively, were obtained, in the identification of patients with breast cancer, indicating that PLIN1 is indeed an excellent marker for human breast cancer.

We further performed a meta-analysis of the prognostic significance of PLIN1 expression in human breast cancer patients (Figure 2D–2E). A total of 24 studies (pools) were used for metastatic relapse (MR) (Supplementary Table 2) and 32 studies (pools) were used for any event (AE) meta-analysis (Supplementary Table 3). The total number of patients included was 3,826 and was 5,041 for MR and AE, respectively. A Univariate Cox proportional hazards model analysis was subsequently performed (Supplementary Table 4 and Supplementary Table 5). The MR (*p* = 0.0003, HR = 0.89, 95% CI = 0.84–0.95) and AE (*p* = 0.0006, HR = 0.92, 95% CI = 0.87–0.96) data indicated that low PLIN1 expression is associated with poor prognosis for breast cancer. The results also indicated that patients with low PLIN1 mRNA expression levels exhibited significantly decreased AE- free overall survival (*p* = 0.0005) (Supplementary Figure 3B, red dashed).

### PLIN1 is an independent marker of disease outcome in ER-positive and luminal A patients

**Figure 3:**
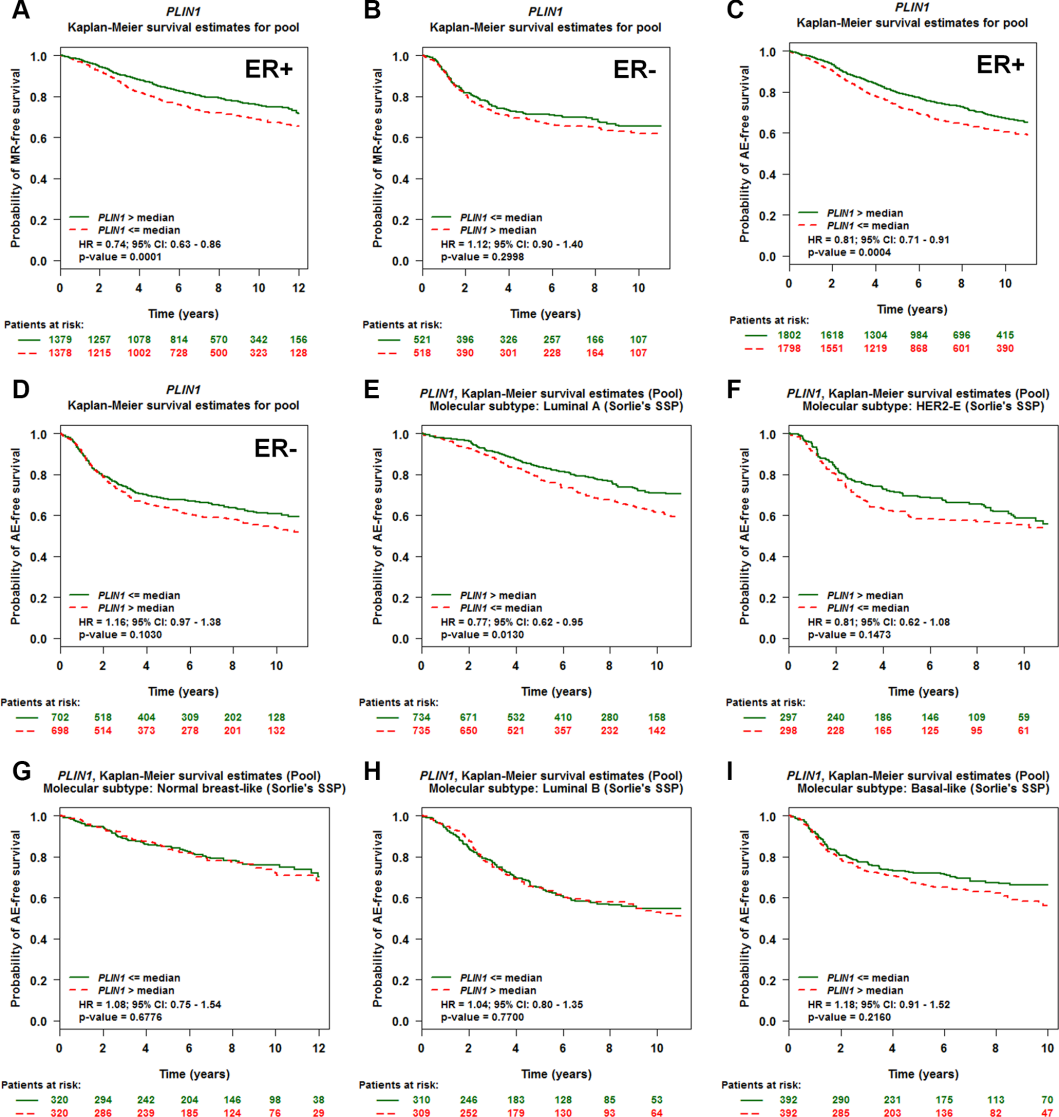
Evaluation of PLIN1 as an independent marker for disease outcome in breast cancer patients with different ER statuses and molecular subtypes (**A–B**) Kaplan-Meier MR-free survival curves for PLIN1 in ER-positive (A, *N* = 2,757) and ER-negative (B, *N* = 1,039) patients. (**C–D**) Kaplan-Meier AE-free survival curves for PLIN1 in ER-positive (*N* = 3,600) (A) and ER-negative (*N* = 1,400) patients. (**E–I**) Kaplan-Meier AE-free survival curves for PLIN1 within the breast cancer molecular subtypes, including luminal A (E, *N* = 1,484), HER2-E (F, *N* = 601), normal breast-like (G, *N* = 653), luminal B (H, *N* = 627) and basal-like (I, *N* = 790) subtypes.

Because estrogen receptor (ER) and nodal status in breast cancer are important prognostic indicators of recurrence and greatly influence treatment regimens [31, 32], we next set out to identify the prognostic potential of PLIN1 expression in breast cancer patients with different ER and nodal statuses. We therefore performed a series of Univariate Cox proportional hazards model analyses on each of the 18 pools corresponding to a combination of (ER and nodal status) populations and event (MR or AE) criteria (Table 1). These results indicate that PLIN1 expression in ER-positive patients exhibits significant prognostic significance (for NM, ER+ and AE: *p* value < 0.0001, HR = 0.87, 95% CI = 0.81–0.92, NP = 3,600), whereas it does not in ER-negative patients (*p* = 0.08, HR = 1.08, 95% CI = 0.98–1.20, NP = 1,039). In contrast, nodal status exhibited no correlation. Thus, we further generated Kaplan-Meier curves based on the ER status. Low PLIN1 expression levels correlated with both shorter MR-free and AE-free survival only among the ER- positive (ER+) patients (Figure 3A–3B), but not among ER- negative (ER−) patients (Figure 3C–3D).

**Table 1:** Target prognostic analysis for the PLIN1 expression level in 18 pools corresponding to combinations of populations (ER and nodal status) and event criteria (MR or AE)

Nodal status	Estrogen receptor status	Event status	*p* value	HR	95% CI	No. patients	No. events
NM	ER+	AE	< 0.0001	0.87	0.81–0.92	3,600	11
NM	ER+	MR	< 0.0001	0.84	0.77–0.90	2,757	658
N−	ER+	MR	0.0001	0.79	0.70–0.89	1,389	312
NM	ERM	MR	0.0003	0.89	0.84–0.95	3,826	993
NM	ERM	AE	0.0006	0.92	0.87–0.96	5,041	1,636
N−	ER+	AE	0.0009	0.85	0.77–0.93	1,744	492
N−	ERM	MR	0.006	0.88	0.80–0.96	1,887	454
N−	ERM	AE	0.01	0.91	0.84–0.98	2,404	711
N+	ER+	AE	0.02	0.89	0.80–0.98	1,023	389
N+	ER+	MR	0.03	0.86	0.75–0.98	677	202
N+	ERM	MR	0.04	0.89	0.79–0.99	980	322
N+	ERM	AE	0.06	0.93	0.86–1.00	1,470	60
NM	ER−	AE	0.08	1.08	0.99–1.17	1,400	525
NM	ER−	MR	0.14	1.08	0.98–1.20	1,039	330
N−	ER−	MR	0.14	1.13	0.96–1.33	479	140
N−	ER−	AE	0.27	1.08	0.94–1.23	635	2
N+	ER−	AE	0.56	1.04	0.91–1.19	438	212
N+	ER−	MR	0.73	1.03	0.86–1.25	295	119

N (+,−, m): nodal status (+: positive, −: negative, m: mixed); estrogen receptor status (+: positive, −: negative, m: mixed); HR: hazard ratio (values are rounded to 2 decimal places); 95% CI: 95% confidence interval (values were rounded to 2 decimal places); ER (+,−, m).

We next set out to assess the prognostic utility of PLIN1 expression in predicting disease outcomes within the individual molecular subtypes, which were classified as normal breast-like, luminal A, luminal B, HER2-E (HER2-enriched) and basal-like subtypes based on PAM50 [33]. A total of 4,155 breast cancer patients with any event information (metastasis, relapse, or death) for the molecular subtype prognostic analyses were used (Supplementary Table 6). Although the expression level of PLIN1 was significantly increased among the luminal A and normal breast-like subtypes (Supplementary Figure 3D–3E), PLIN1 expression levels among the luminal A subtypes correlated with more favorable prognosis (*p* = 0.02, HR = 0.88, 95% CI = 0.78–0.98, NP = 1,469) compared to those observed in the other four subtypes based on Sorlie's classification [34]. In addition, patients with low expression of PLIN1 exhibited a reduced AE-free survival time among luminal A subtypes compared to basal-like, luminal B and HER2-E subtypes (Figure 3E–3I).

### Validation of the PLIN1 expression in breast cancer tissues

**Figure 4:**
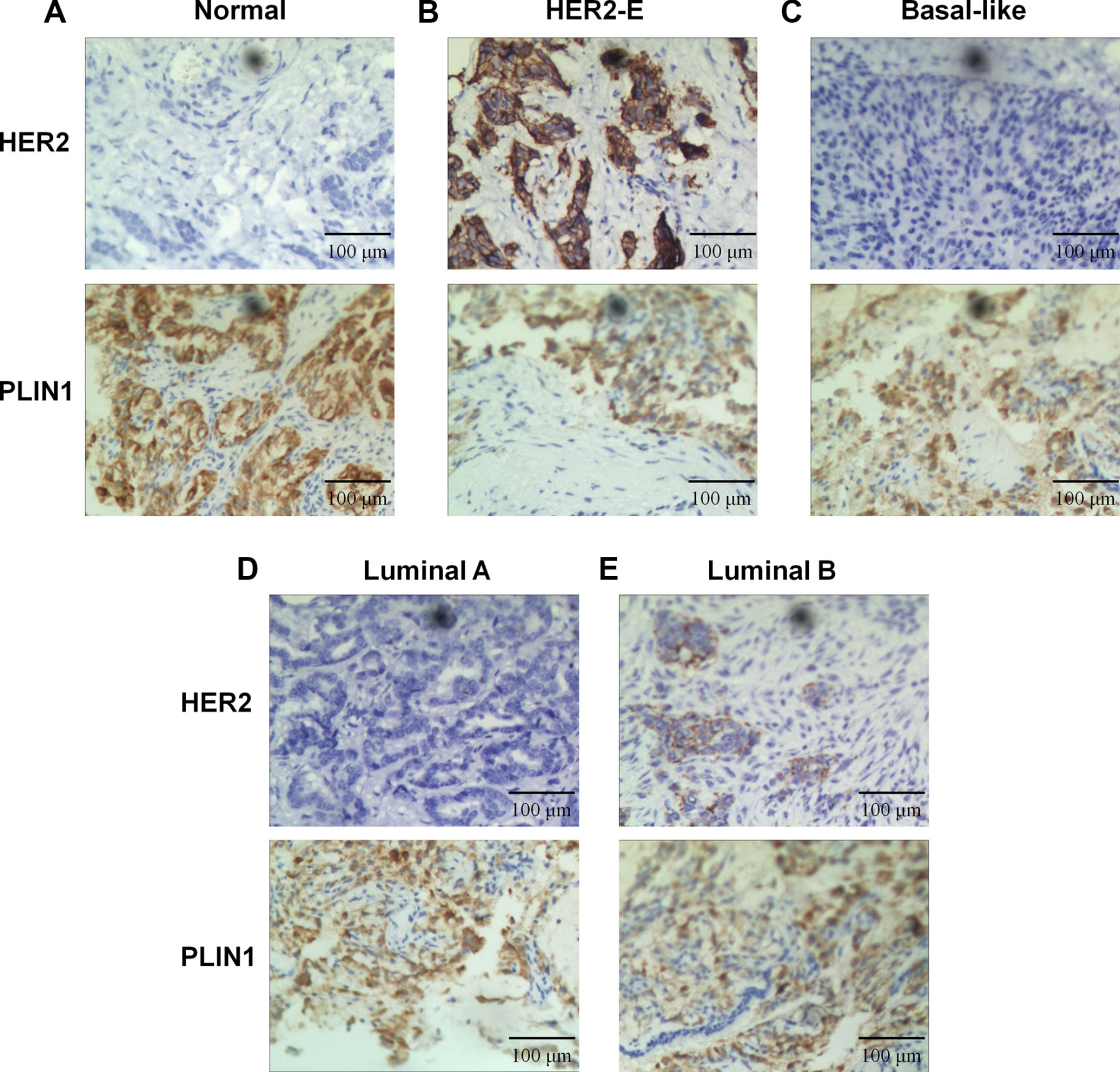
Expression of PLIN1 in breast cancer Compared with normal tissue (**A**), immunohistochemical staining revealed a significantly reduced staining area of PLIN1 in HER2-E (**B**), basal-like (**C**), luminal A (**D**) and luminal B (**E**) subtypes. HER2 staining was used as a control.

To validate PLIN1 expression in breast cancer, we performed immunohistochemical analysis on a total of 40 pairs of human breast cancer tissues (10 pairs for each subtype). The expression of PLIN1 was semi-quantitatively assessed based on the total staining intensity and percentage of nuclear and cytoplasmic staining of PLIN1. We predominantly detected PLIN1 within the cytoplasm and to a significantly lesser extent, within the nucleus (Figure 4). PLIN1 was downregulated across all subtypes compared with the normal control samples, consistent with PLIN1 expression trends from the TCGA database. Furthermore, we found that low expression of PLIN1 in human brain glioma significantly correlates with the WHO classification (*p* < 0.01), which signifies that decreased PLIN1 occurs more frequently in advanced tumors.

### PLIN1 inhibits breast cancer cell proliferation, migration and invasion

**Figure 5:**
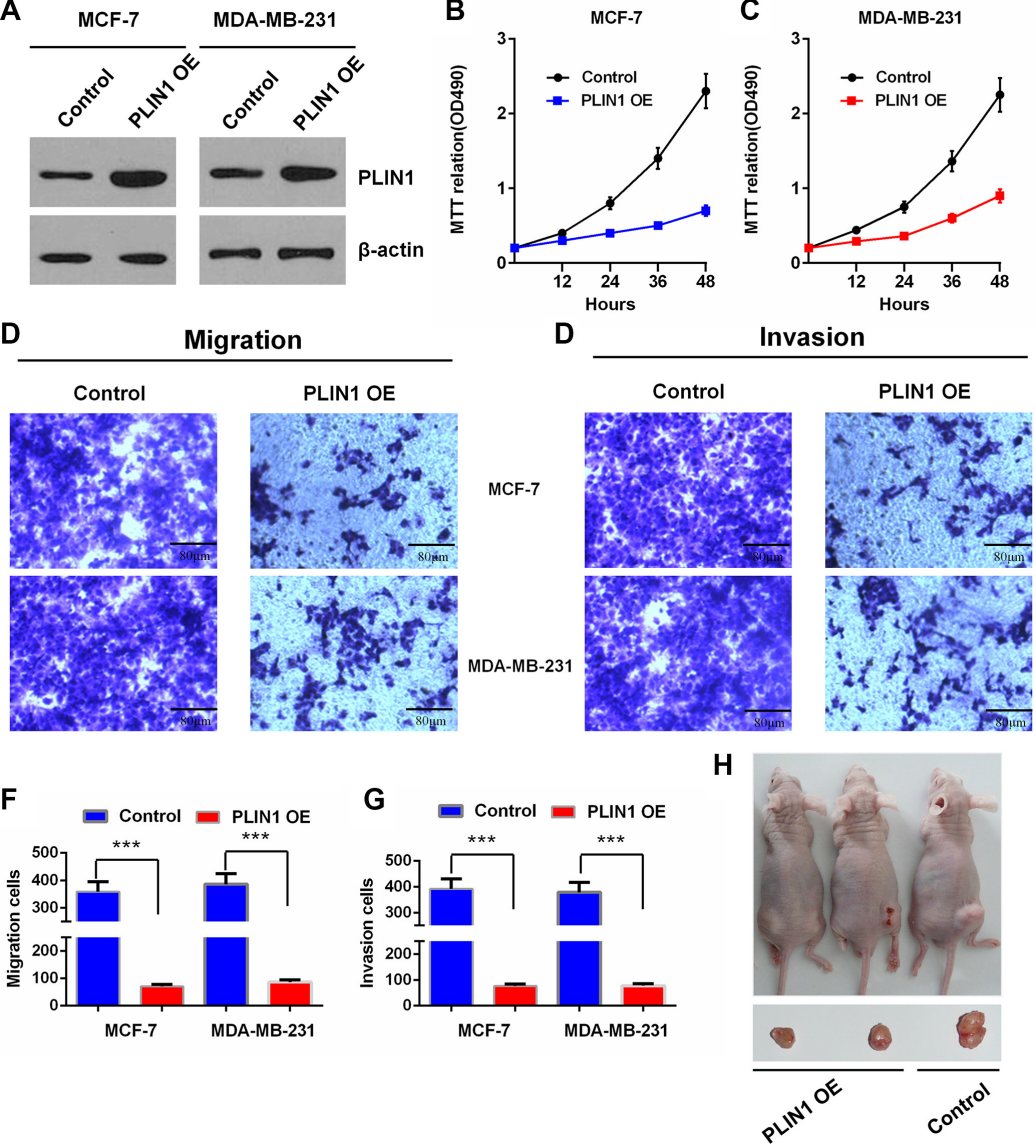
Effects of PLIN1 on human breast cancer cell proliferation, migration and invasion (**A**) Western blot assay shows the PLIN1 expression levels after transfection with the PLIN1-expressing recombinant plasmids in MCF-7 and MDA-MB-231 cells. (**B–C**) Cell proliferation analysis by MTT assays for MCF-7 (B) and MDA-MB-231 cells (C) with or without exogenous PLIN1. (**D–G**) Transwell assays show the effects of PLIN1 on MCF-7 and MDA-MB-231 breast cancer cell migration and invasion. Representative micrographs and statistical data exhibit the effects of PLIN1 on cell migration (D and F) and invasion (E and G). The data are presented as the mean values ± SD. The Two-tailed Student's *t*-test was used. *p < 0.05, **p < 0.01 and ***p < 0.001. (**H**) Representative pictures from a total of 6 tested and 6 control mice, showing tumorigenesis of hind limbs isolated from nude mice three weeks after injection of cells stably expressing PLIN1 or control cells.

As PLIN1 exhibits significant prognostic significance in human breast cancers, we investigated the functional role of PLIN1 in breast cancer. We first examined the effects of exogenous PLIN1 on the proliferation of the human breast cancer cell line MCF-7. Western blotting analysis verified the expression efficiency of an exogenous PLIN1 expression plasmid (Figure 5A). We next performed MTT assays and found that the proliferation of MCF-7 cells was significantly decreased by exogenous expression of PLIN1. Consistent results were observed in MDA-MB-231 cells (Figure 5B–5C).

Human breast cancer cell migration and invasion were further evaluated by the examining the effects of exogenous PLIN1. Transwell assays were used to determine the effect of PLIN1 on cell migration. To this end, we incubated MCF-7 and MDA-MB-231 cells in transwell chambers for 6 hours before counting cells that crossed the insert (see Methods). We found that compared with control cells, cells transfected with exogenous PLIN1 exhibited a significantly decreased migratory ability (Figure 5D and 5F). To examine the effect of PLIN1 on cell invasion, we cultured MCF-7 and MDA-MB-231 cells transfected with exogenous PLIN1 in transwell chambers pre-coated with matrigel for 8 hours prior to measurements. We found that increased PLIN1 expression significantly decreases the ability of the cells to cross the matrigel-coated inserts (Figure 5E and 5G).

To evaluate the effect of exogenous PLIN1 expression on the breast tumor growth *in vivo*, we established PLIN1-transfected stable or control MDA-MB-231 cells and injected them subcutaneously into nude mice. Cells with PLIN1 over-expression produced much larger and faster growing breast tumors compared with control cells (Figure 5H). Taken together, our data demonstrate that high PLIN1 levels significantly inhibit human breast cancer cell proliferation, invasion, migration, and *in vivo* tumorigenesis.

### Correlation of PLIN1 expression with disease outcome in other human cancer types

To investigate whether the downregulation of PLIN1 might contribute to the pathogenesis of other cancers, the mRNA levels of PLIN1 in several human cancers was assessed using the cBioPortal database. The results revealed that PLIN1 expression is shallow deleted in 21 human cancer types, of which 14 exhibited deletion of PLIN1 in more than 15% (66.7%, 14/21) of cases, 7 exhibited deletion in more than 20% (33.3%, 7/21) of cases and 3 exhibited deletion in more than 30% (14.3%, 3/21) of cases (Supplementary Figure 4A). The PLIN1 mRNA levels were significantly reduced in tumors with a shallow deletion of PLIN1 compared to those without such changes, including low-grade glioma, breast cancer, cervical squamous cell carcinoma, liver hepatocellular carcinoma, lung adenocarcinoma, lung squamous cell carcinoma, pancreatic adenocarcinoma and sarcoma (*p* < 0.05, Supplementary Figure 4B and Table 2). These findings suggest that PLIN1 deletion results in the reduced expression of PLIN1 in the above-mentioned cancers. However, patients with PLIN1 amplification also exhibited low PLIN1 expression in low-grade glioma and pancreatic adenocarcinoma (*p* < 0.05).

**Table 2:** Correlation of PLIN1 expression with copy number changes in different cancer types

Cancer type	Shallow deletiion	Diploid	Gain	*p* value (shallow deletion vs diploid)	*p* value (gain vs diploid)
Colorectal Adenocarcinoma	123	229	22	0.56	0.20
Adrenocortical Carcinoma	17	48	10	0.82	0.28
Brain Lower Grade Glioma	54	445	13	0.0003	0.006
Breast Invasive Carcinoma	270	626	147	< 0.0001	< 0.0001
Cervical Squamous Cell Carcinoma	44	175	64	0.001	0.24
Glioblastoma Multiforme	28	108	10	0.91	0.22
Esophageal Carcinoma	45	86	45	0.54	0.15
Stomach Adenocarcinoma	4	21	6	ND	ND
Uveal Melanoma	4	74	2	ND	ND
Head and Neck Squamous Cell Carcinoma	98	327	87	0.57	0.92
Kidney Renal Clear Cell Carcinoma	36	461	27	0.17	0.27
Liver Hepatocellular Carcinoma	71	247	46	< 0.0001	0.88
Lung Adenocarcinoma	210	238	60	0.0002	0.40
Lung Squamous Cell Carcinoma	104	232	151	0.03	0.09
Ovarian Serous Cystadenocarcinoma	128	113	45	0.16	0.28
Pancreatic Adenocarcinoma	29	128	14	0.002	0.004
Mesothelioma	6	64	15	ND	0.04
Prostate Adenocarcinoma	26	298	8	0.47	ND
Skin Cutaneous Melanoma	40	209	111	0.60	0.58
Sarcoma	36	125	68	< 0.0001	0.15

ND: not done, because of no cases or a small number of cases (< 10).

**Figure 6:**
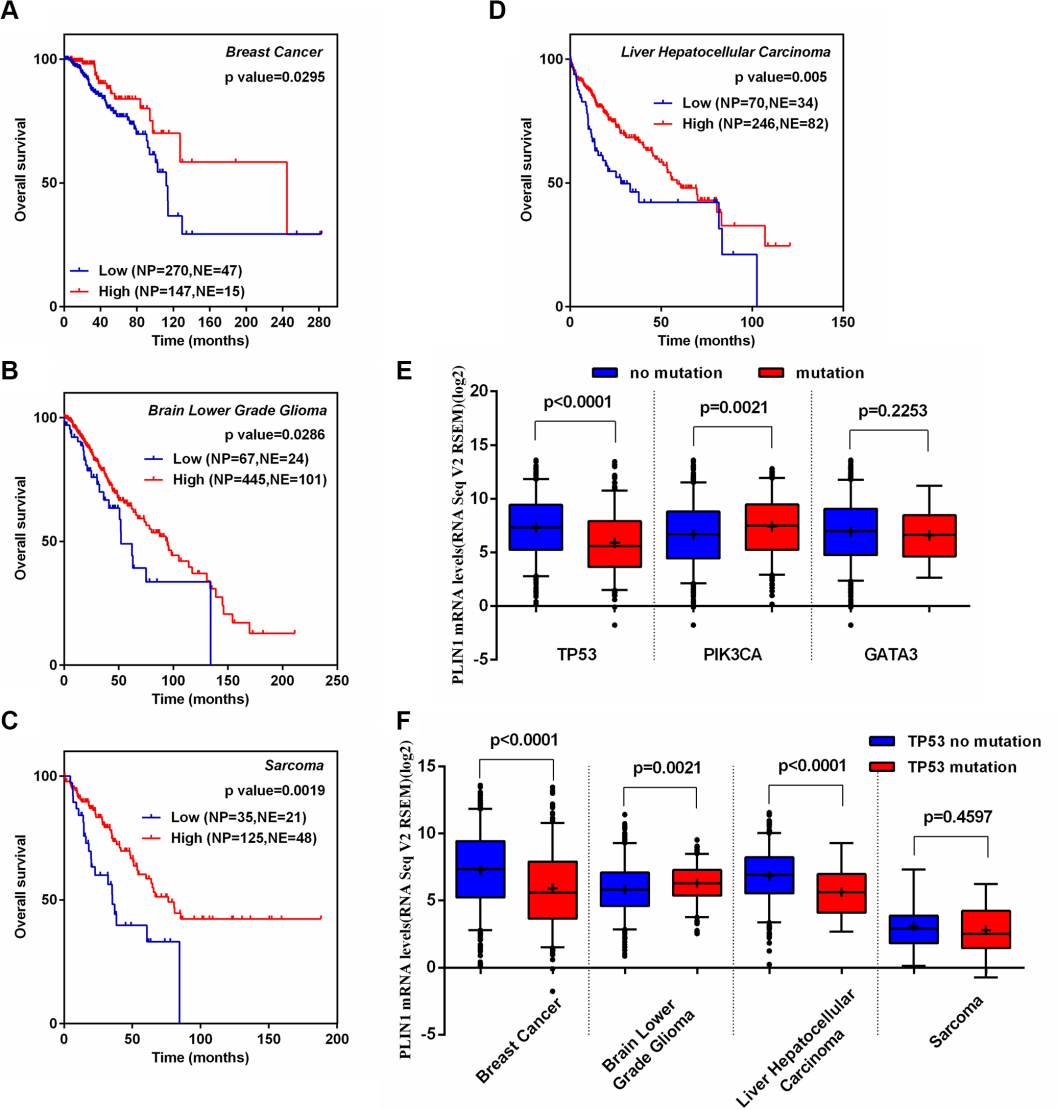
Correlation of PLIN1 expression with disease outcome in several human cancer types and TP53 somatic mutations (**A–D**) Kaplan-Meier survival curves for the patients with low or high levels of PLIN1 in breast cancer (A), low-grade glioma (B), sarcoma (C) and hepatocellular carcinoma (D). (**E**) Association of PLIN1 expression with somatic TP53, PIK3CA and GATA3 mutations in breast cancer. (**F**) Association of PLIN1 expression with somatic TP53 mutations in 3 other human cancers. All datasets were obtained from cBioPortal.

We further generated several Kaplan-Meier analysis curves based on the cBioPortal database for 8 cancers types, in which low PLIN1 expression correlated with PLIN1 deletion, as detailed above. We found that breast cancer patients with low PLIN1 mRNA had have significantly reduced overall survival (Figure 6A, *p* = 0.03), which is consistent with our meta-analyses of the microarray datasets. Furthermore, the low PLIN1 mRNA levels also correlated with decreased overall survival in the other 3 human cancer types, including low-grade glioma, liver hepatocellular carcinoma and sarcoma (Figure 6B–6D and Supplementary Figure 5).

### Association of PLIN1 expression with TP53 DNA somatic mutations

Notably, somatic mutations in cancer driver genes are unevenly distributed across tumor subtypes. Somatic mutations in TP53, PIK3CA and GATA3 occurred at a > 10% incidence rate across all breast cancers [35]. Thus, we investigated the potential relationship between PLIN1 with mutated TP53, PIK3CA and GATA3 in breast cancer. We found that TP53 mutations correlated with a significant reduction in PLIN1 expression (*p* < 0.001). In contrast, mutant PIK3CA exhibited a significant increase (*p* = 0.002) (Figure 6E), suggesting that TP53 and PIK3CA might contribute to the regulation of PLIN1 expression respectively, of which TP53 plays the major role. We further investigated whether mutant TP53 decreases PLIN1 mRNA expression in low-grade glioma, liver hepatocellular carcinoma and sarcoma. Our data showed consistent results in hepatocellular carcinoma as in breast cancer (Figure 6F). Taken together, these data suggest that mutant TP53 contributes to low PLIN1expression in breast cancer and hepatocellular carcinoma.

## DISCUSSION

In this study, we identified 57 genes that exhibit deregulated expression patterns in breast cancer using patient expression data obtained from the TCGA database. Gene Ontology annotation and KEGG pathway analysis revealed that these target genes are predominantly enriched in the regulation of lipid metabolic process. Four deregulated genes in breast cancer patients were further identified and exhibited significant correlation with overall survival. Among these genes, PLIN1 exhibited a particularly significant correlation.

By meta-analysis of public microarray profiles, we confirmed the prognostic value of PLIN1 expression in breast cancer patients. Our results indicate that patients with low PLIN1 mRNA levels have decreased overall and MR-free survival time, particularly in ER-positive and luminal A subtype patients. From the PLIN1 expression map of RSSPC classifications (Supplementary Figure 3E), we found the lowest levels of PLIN1 expression were exhibited in luminal B and basal-like subtype samples, which have a very low level of HER2 differs from the other three subtypes (Figure 5). Because HER2 plays an important role in the development and progression of breast cancer by mediating multiple signals in cancer cells [36], our findings suggest that HER2 status is involved in the downregulation of PLIN1 mRNA expression. Using the TCGA database, we also confirmed that reduced levels of PLIN1 expression correlate with significantly reduced overall survival rates.

The expression levels of PLIN1 were further verified by immunohistochemical assessment in breast cancer tissues, and the resulting data were consistent with those from the RNA- sequencing and microarray assays. Notably, the exogenous expression of PLIN1 in human breast cancer cell lines MCF-7 and MDA-MB-231 significantly inhibited cellular proliferation, migration and invasion. Combining with the role of PLIN1 in lipid droplet biogenesis, our data suggest a crucial role of PLIN1-mediated lipid metabolism in the tumor development of breast cancer, which is fundamentally a disorder of cell growth and proliferation and requires cellular building blocks, such as nucleic acids, proteins and lipids [37]. Thus, the role of PLIN1 in human breast cancer warrants further investigations.

Somatic mutations in cancer driver genes are unevenly distributed across tumor subtypes and TP53 is one of the top three (TP53, PIK3CA and GATA3) most frequently mutated genes in breast cancer. We found that TP53 mutations coincide with a decrease in the expression of PLIN1 in tumor compared with normal control samples. Furthermore, TP53 mutations are overwhelmingly enriched in HER2-enriched and basal-like subtypes [35]. Our results from the PLIN1 expression map suggest a relationship between TP53 mutations and HER2 status that is involved in the downregulation of the PLIN1 mRNA expression. Moreover, we also evaluated a probable phenomenon in hepatocellular carcinoma. Although the role of PLIN1 in liver hepatocellular carcinoma was previously unknown, Lipid droplet proteins (PLIN1-PLIN5) have been previously reported as being involved in the pathophysiology of fatty liver diseases that are characterized by excessive lipid accumulation in hepatocytes [38], as well as in liver steatosis [39]. The diagnosis and prognosis value of PLIN1 in hepatocellular carcinoma and the involved regulatory mechanism warrants in-depth study.

Overall, we identify PLIN1 as a potential biomarker for multiple human cancer types, including breast cancer, low-grade glioma, hepatocellular carcinoma and sarcoma and highlight the prognostic value of PLIN1 mRNA levels in breast cancer outcomes. These results suggest that PLIN1 is involved in breast cancer progression and might act as a tumor suppressor gene. However, further studies are needed to enhance our understanding of the mechanistic roles of PLIN1 in the development and progression of breast cancer.

## MATERIALS AND METHODS

### Clinical specimens

A total of 40 pairs of human breast cancer tissues were collected from Hubei General Hospital (Renmin Hospital of Wuhan University, Hubei, China), and Tongji Hospital (Hubei, China) between 2013 and 2015 during surgery and made into paraffin sections (4 μm). No enrolled patients underwent radiation or chemotherapy prior to surgery. All specimen collections and studies thereof were approved by the Ethics Committee of the source hospitals and all patients provided the written consensus for this study. All experiments were performed in accordance with principles expressed in the Declaration of Helsinki or other relevant guidelines and regulations.

### Cell culture and transfection

The human breast cancer cell lines MCF-7 and MDA-MB-231 were purchased from the Cell Center of Institute of Biochemistry and Cell Biology, Chinese Academy of Sciences (Shanghai, China). The cells were cultured in Dulbecco's modified Eagle's medium (DMEM) (Gibco, USA) supplemented with 10% fetal bovine serum (Gibco, USA), 100 U/ml penicillin G and 100 μg/ml streptomycin at 37°C in a humidified incubator containing 5% CO2. Lipofectamine 2000 Transfection Reagent (Invitrogen, USA) was used to transfect the MCF-7 and MDA-MB-231 cell lines with the PLIN1-expressing recombinant plasmid (p3xflag-cmv-10) according to the manufacturer's protocols.

### Western blotting and immunofluorescence

Total proteins from MCF-7 and MDA-MB-231 cell lysates were extracted by resuspending the cell pellets in RIPA buffer (150 mM NaCl, 50 mM Tris (pH 7.4) and 1% Triton X-100). Approximately 55 μg of total protein per sample was separated by SDS-PAGE and then transferred onto nitrocellulose membranes. Western blot analyses were performed with polyclonal antibodies against PLIN1 (Santa Cruz Biotechnology, USA), with a monoclonal β-actin antibody as a control (Sigma, USA).

### Immunohistochemistry

Immunohistochemistry was performed as previously described [40]. Briefly, paraffin sections were deparaffinized successively in 100% xylene, 95% alcohol, 90% alcohol, 80% alcohol and 70% alcohol and then rehydrated for 10 minutes. Next, hydrogen peroxide (0.3% v/v) was applied to block endogenous peroxide activity and the samples were microwave heated in 15 μM citrate buffer (pH 6.0) for 3 minutes to expose the antigens. The paraffin sections were then incubated with normal goat serum to reduce non-specific antibody binding. Next, the tissue sections were incubated with a PLIN1 polyclonal antibody (1:1,000 dilutions, Santa Cruz Biotechnology). Rabbit immunoglobulin (1:1,000 dilutions) was used as a negative control. Antibody staining was performed by overnight incubation at 4°C with gentle shaking. Next, the samples were incubated with the secondary biotinylated goat anti-rabbit serum immunoglobulin G (IgG) antibody at 37°C for 30 minutes. After washing, the paraffin sections were incubated with streptavidin-avidin-conjugated horseradish peroxidase for 30 minutes. Counterstaining with hematoxylin was performed for 30 minutes and the paraffin sections were dehydrated in ethanol prior to mounting.

### MTT assay

Cells (1 × 10^5^ cells/well) were seeded into 6-well plates. Cell proliferation was examined at 12, 24, 36 and 48 hours after transfection. The cells were stained at the indicated time points with 100 μl of sterile MTT dye (0.5 mg/ml, Sigma, USA) for 4 hours at 37°C, followed by removal of the culture medium and the addition of 150 μl of DMSO (Sigma). The number of viable cells was assessed by measurement of the absorbance at 490 nm. All experiments were performed in triplicate.

### Cell migration and invasion assays

For cell migration assays, 1 × 10^4^ cells in 100 μl of DMEM without fetal bovine serum were seeded onto fibronectin-coated polycarbonate membrane inserts in transwell chambers (Costar Corning, USA). In the lower chamber, 500 μl of DMEM supplemented with 10% fetal bovine serum was added as a chemoattractant. After the cells were incubated for 6 hours at 37°C with 5% CO2, the inserts were washed with phosphate buffered saline and the cells on the top surface of the inserts were removed with a cotton swab. Cells adhering to the lower insert membrane surfaces were fixed with methanol stained with crystal violet solution and quantified using ImageJ software. All assays were independently repeated in triplicates. The procedure for cellular invasion assays was similar to that of the cell migration assays, except that the transwell membranes were precoated with 24 μg/μl matrigel (R&D Systems Inc., USA) and that the cells were incubated for 8 hours at 37°C with 5% CO2. Cells adhering to the lower insert membrane surfaces were counted in the same way as in the cell migration assays.

### *Xenograft* tumorigenesis assay using nude mice

12 nude mice (8–10 weeks old) were obtained from Animal Biosafety Level III Laboratory at the Center for Animal Experiments, Wuhan University (Wuhan, China) and divided into two groups (6 each). They were subcutaneously injected with 5.5 × 10^6^ PLIN1-transfected stable or control MDA-MB-231 cells per mouse. Hind limbs from both groups were harvested and photographed three weeks later.

### The cancer genome atlas (TCGA) analysis of gene expression in breast cancer patients

Breast cancer UNC IlluminaHiSeq_RNASeq Level 3 data were downloaded from the TCGA data portal (http://cancergenome.nih.gov/). Gene expression was quantified as fragments per kilo base transcript per million mapped reads (FPKM). The “gene.quantification“result files (*N* = 307) were used without further normalization. The RNAseq data were grouped into Tumor tissues (*N* = 208) and Normal tissues (*N* = 99) based on TCGA annotation. The heatmap analysis of the gene expression pattern was performed by R version 3.2.2 software for Windows with “DESeq” and “edgeR” packages. Genes were hierarchically clustered using complete linkage and Euclidian distance. Fold-change analysis was performed on the two categories of samples (Normal and Tumor), followed by an unpaired *t*-test (unequal variance) that was performed to obtain significant gene entities. The *p*-value computation (asymptotic) was further performed to obtain gene entities with *p* < 0.01 and log2fold-change (log2FC) > 4.0. The concordant gene entities were identified across the two packages by using Microsoft Access.

### Datasets used for functional annotation

The concordant gene list across the two packages was analyzed using different web resources. Gene Ontology Analysis was performed using the DAVID (Database for Annotation, Visualization and Integrated Discovery) classification system (https://david.abcc.ncifcrf.gov/) to evaluate the functional classes of the genes. The STRING database version 10 (Search Tool for the Retrieval of Interacting Genes/Proteins) (http://string-db.org/) was used to predict and catalog the protein-protein interactions between the concordant genes. Genomic alterations and mRNA expression levels, TP53 mutations, fraction of copy number alteration, frequency of gene mutations and clinical information for the set of samples were obtained from cBioPortal (http://www.cbioportal.org/data_sets.jsp). Bc-GenExMiner v3.2 (http://bcgenex.centregauducheau.fr/BC-GEM/GEM_Requete.php?js=1) were used to performed the meta-analysis for breast cancer AE survival, MR-free survival, breast cancer subtype and breast cancers with clinicopathological information in breast cancer datasets.

### Statistical analysis

Receiver operating characteristic (ROC) curve analysis was used to evaluate the predictive power of each biomarker. The area under the curve (AUC) was computed via numerical integration of the ROC Curves. The median cut was used in all survival analyses and log rank *p*-values were calculated. Group comparisons were performed using the Mann-Whitney test and the two-tailed *p*-value is shown. Survival analysis and ROC curves were performed using GraphPad Prism version 6.01. Cox proportional hazard models analysis was used to calculate hazard ratios and to identify factors affecting survival. All analyses were performed by SPSS 13.0 for windows. A two-tailed *p*-value of less than 0.05 was considered statistically significant.

## SUPPLEMENTARY MATERIALS




