# Supplementary material for: Prognostic significance of PLIN1 expression in human breast cancer
Source: Oncotarget. 2016 Jun 23;7(34):54488–502. doi: 10.18632/oncotarget.10239 (PMC5342357; doi:10.18632/oncotarget.10239)
Supplement: Supplementary file 1 [file oncotarget-07-54488-s001.pdf]

Prognostic significance of PLIN1 expression in human breast cancer

Supplementary Materials

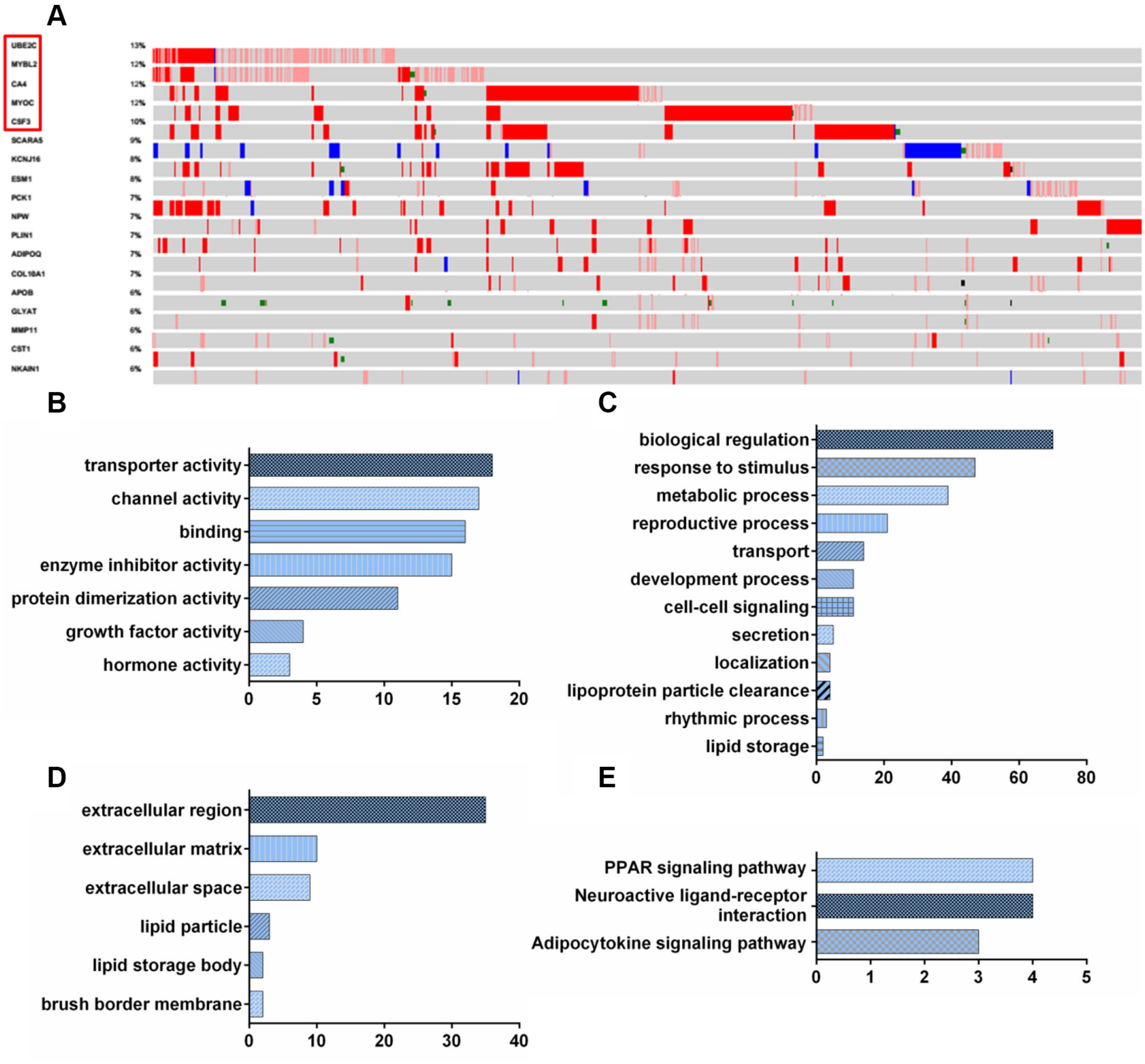

Supplementary Figure S1: Gene ontology analyses of the concordant genes. (**A**) The concordant, significant gene entities were analyzed for mutation, copy number variation (CNV) and gene expression alteration status in TCGA human breast cancer patient cohorts. The genes, which was framed including UBE2C, MYBL2, CA4, MYOC, CSF3, showed alteration in highest proportion (> 10%) of the patients. (**B–D**) Gene ontology analysis was performed for the concordant gene list in the DAVID database for the classification of the genes. The most enriched and significant GO term for the molecular function category was transporter activity (B), for the biological process category was biological regulation (C) and for the cellular components category was extracellular (D). (**E**) KEGG pathway enrichment analysis for the concordant genes.

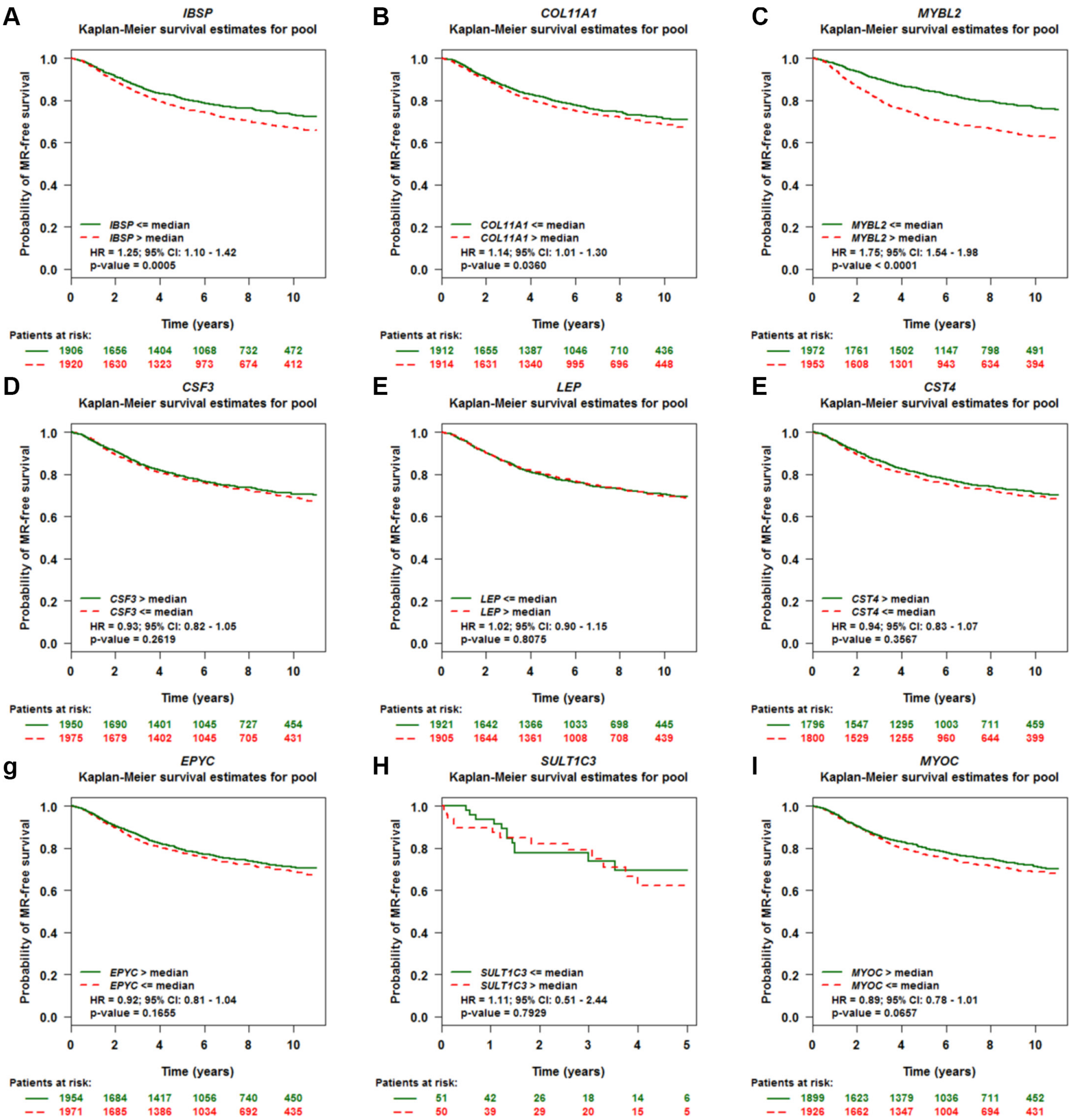

Supplementary Figure S2: Kaplan-Meier MR-free overall survival curves using Bc-GenExMiner v3.2 database for IBSP (A), *N* = 3,826), COL11A1 (B), *N* = 3,826), MYBL2 (C), *N* = 3,925), CSF3 (D), *N* = 3,925), LEP (E), *N* = 3,826), CST4 (F), *N* = 3,596), EPYC (G), *N* = 3,925), SULT1C3 (H), *N* = 101) and MYOC (I), *N* = 3,825).

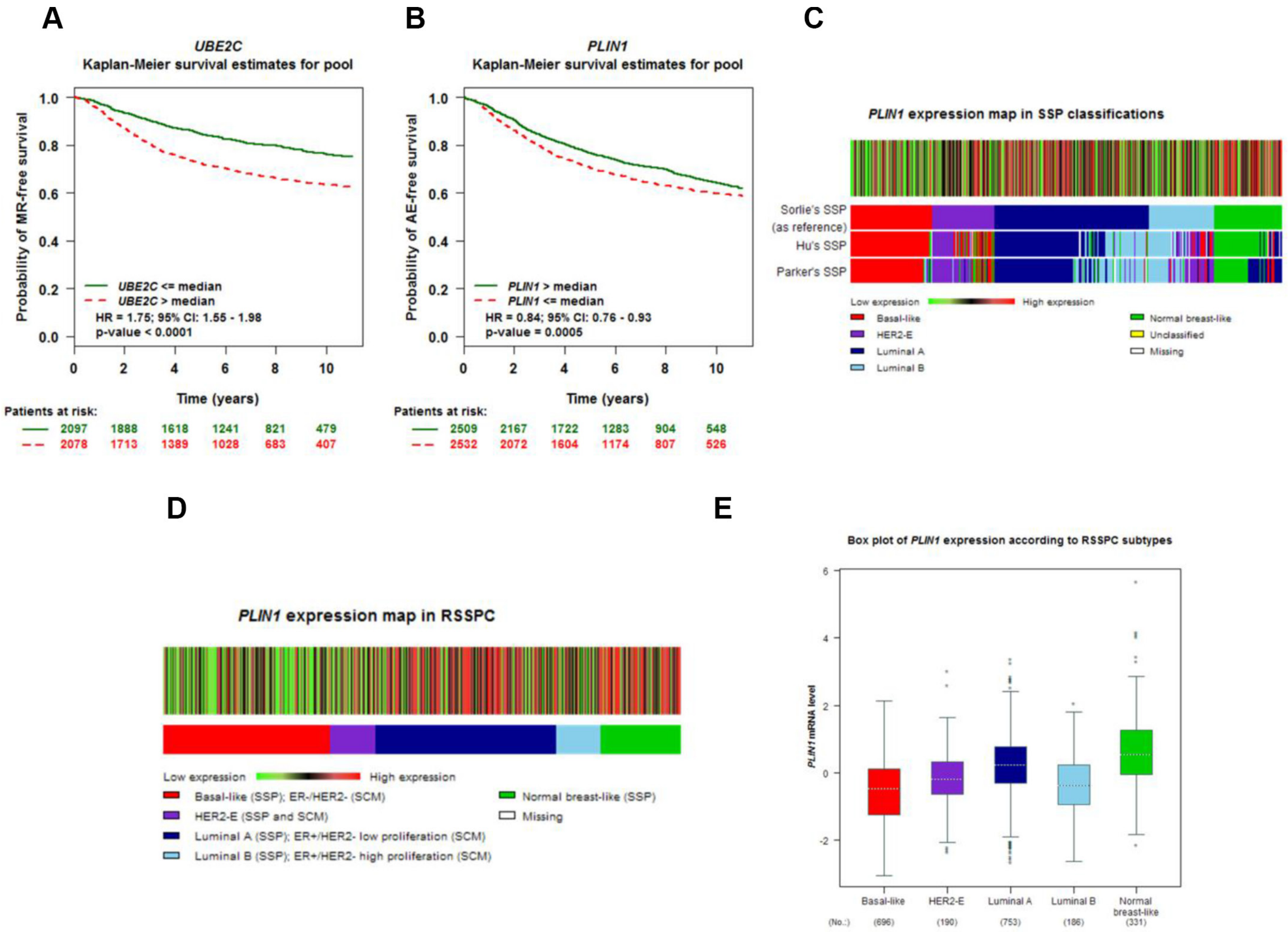

Supplementary Figure S3: (**A**) Kaplan-Meier MR-free overall survival curves using Bc-GenExMiner v3.2 database for UBE2C ($N = 4{,}175$) (**B**) Kaplan-Meier MR-free overall survival curves using Bc-GenExMiner v3.2 database ($N = 5{,}041$). (**C**–**D**) PLIN1expression in different molecular subtypes, classified by SSP (Single Sample Predictor, B) and RSSPC (Robust SSP classification based on patients classified in the same subtype with the three SSPs, C). (**E**) Box plot of PLIN1 expression according to RSSPC subtypes, including basal-like ($N = 699$), HER2-E ($N = 190$), luminal A ($N = 756$), luminal B ($N = 186$) and normal breast-like ($N = 332$).

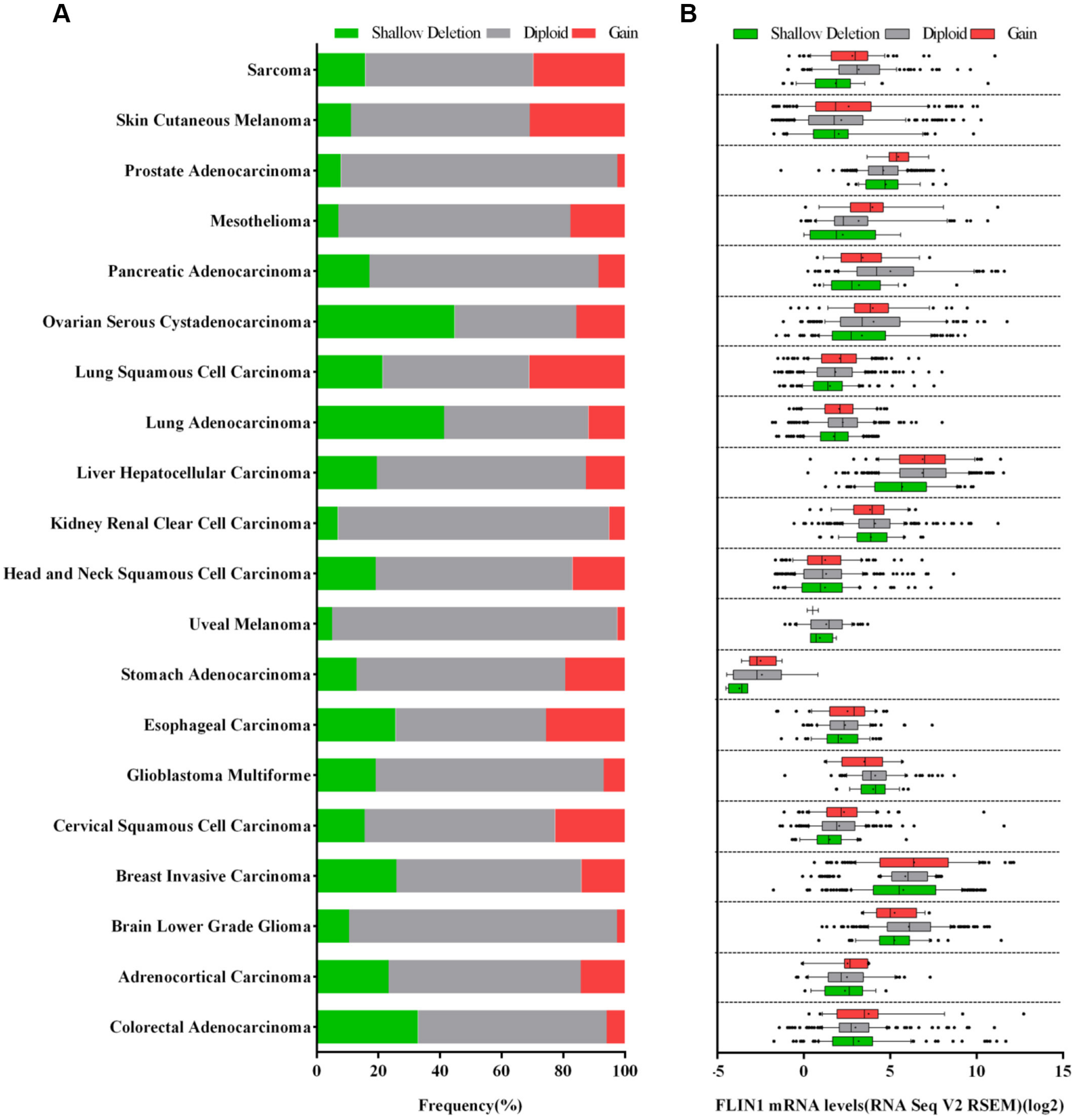

Supplementary Figure S4: Genomic alteration of PLIN1 in several human cancer types. (**A**) Frequencies of PLIN1 genomic alterations in 21 different human cancer types. (**B**) Relationship between PLIN1 mRNA expression levels (log2) and genomic alterations.

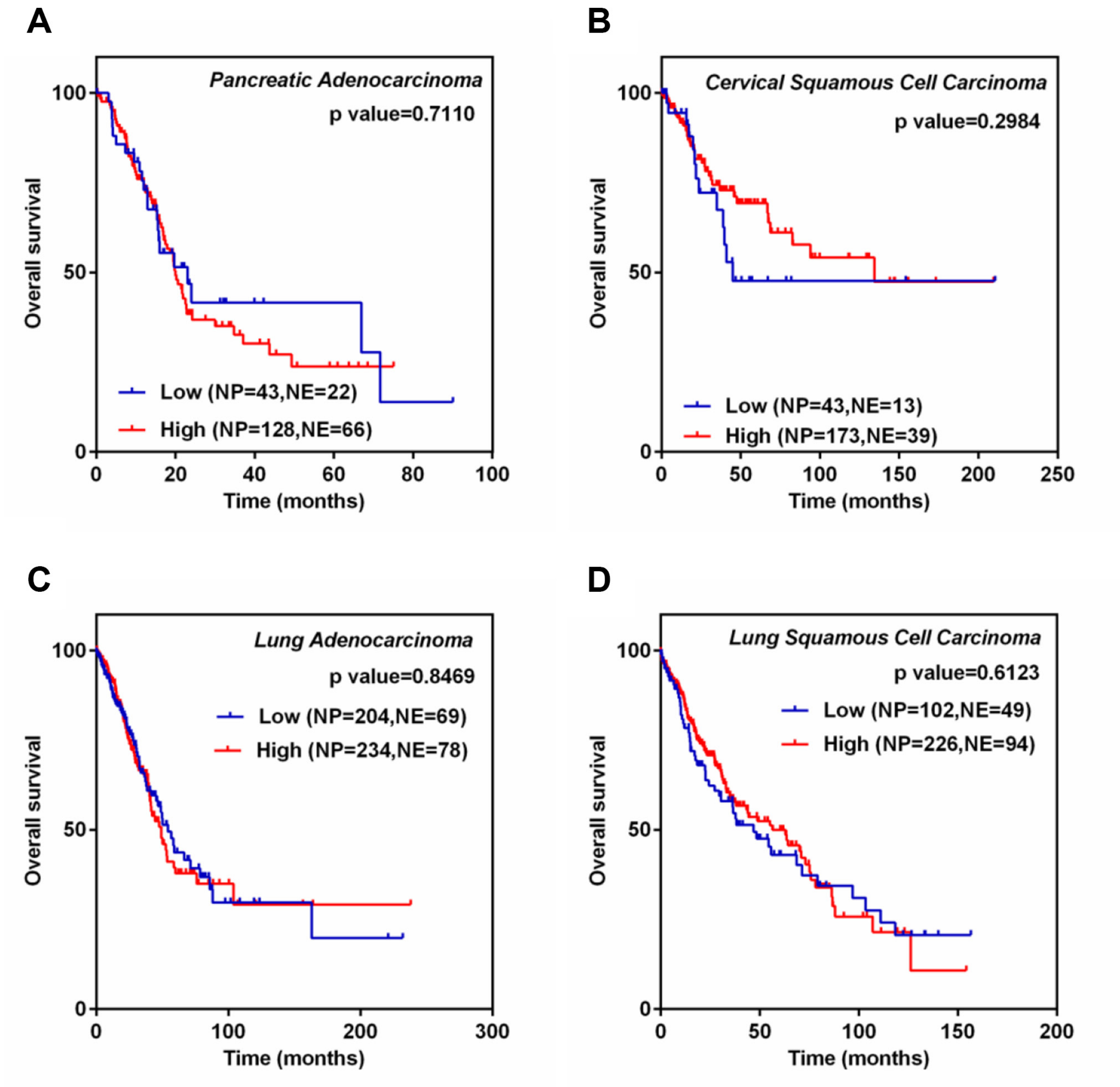

Supplementary Figure S5: Kaplan-Meier survival curves for the patients with low or high PLIN1 in pancreatic adenocarcinoma (A), cervical squamous cell carcinoma (B), lung adenocarcinoma (C) and lung squamous cell carcinoma (D).

Supplementary Table S1: List of concordant differentially expressed genes. See Supplementary_ Table_S1

Supplementary Table S2: Main characteristics and results of the eligible studies for metastatic relapse event

Study code	Original data		Filtered data	Final data	
	Reference	No. patients	(Nm, ERm and MR) -selected patients	No. patients	No. MR
**Rosetta2002**	Van de Vijver et al., 2002 [1]	295	295	295	101
**GSE2603**	Minn et al., 2005[2]	82	82	82	27
**GSE1456**	Pawitan et al., 2005[3]	159	159	159	40
**GSE2034**	Wang et al., 2005[4]	286	286	286	107
**GSE2741**	Weigelt et al., 2005[5]	50	50	50	13
**E_TABM_158**	Chin et al., 2006[6]	112	112	112	21
**GSE8757**	Chin et al., 2007[7]	171	171	171	38
**GSE7390**	Desmedt et al., 2007[8]	198	198	198	62
**GSE6532**	Loi et al., 2007[9]	401	401	393	101
**GSE5327**	Minn et al., 2007[10]	58	58	58	11
**GSE7378**	Zhou et al., 2007[11]	54	54	54	9
**GSE7849**	Anders et al., 2008[12]	75	75	75	14
**GSE9893**	Chanrion et al., 2008[13]	155	155	155	48
**GSE9195**	Loi et al., 2008[14]	77	77	77	10
**GSE11121**	Schmidt et al., 2008[15]	200	200	200	46
**GSE12093**	Zhang et al., 2009[16]	136	136	136	20
**GSE19615**	Li et al., 2010[17]	115	115	115	14
**GSE17907**	Sircoulomb et al., 2010[18]	55	55	39	17
**GSE22219**	Buffa et al., 2011[19]	216	216	216	82
**GSE26971**	Filipits et al., 2011[20]	277	277	258	58
**GSE25055**	Hatzis et al., 2011[21]	309	309	309	65
**GSE20685**	Kao et al., 2011[22]	296	296	296	63
**GSE33926**	Kuo et al., 2012[23]	51	51	51	12
**GSE45255**	Nagalla et al., 2013[24]	41	41	41	14
			**Total:**	**3, 826**	**993**

Supplementary Table S3: Main characteristics and results of the eligible studies for any event

Study code	Original data		Filtered data	Final data	
	Reference	No. patients	(Nm, ERm and AE) -selected patients	No. patients	No. AE
**Rosetta2002**	Van de Vijver et al., 2002 [1]	295	295	295	122
**GSE2603**	Minn et al., 2005[2]	82	82	82	27
**GSE1456**	Pawitan et al., 2005[3]	159	159	159	50
**GSE2034**	Wang et al., 2005[4]	286	286	286	107
**GSE2741**	Weigelt et al., 2005[5]	50	50	50	13
**GSE3143**	Bild et al., 2006[25]	158	158	158	50
**E_TABM_158**	Chin et al., 2006[6]	112	112	112	42
**GSE4922**	Ivshina et al., 2006[26]	249	249	249	89
**GSE8757**	Chin et al., 2007[7]	171	171	171	56
**GSE7390**	Desmedt et al., 2007[8]	198	198	198	91
**GSE6532**	Loi et al., 2007[9]	401	401	393	139
**GSE5327**	Minn et al., 2007[10]	58	58	58	11
**E_UCON_1**	Naderi et al., 2007[27]	135	135	135	65
**GSE7378**	Zhou et al., 2007[11]	54	54	54	9
**GSE7849**	Anders et al., 2008[12]	75	75	75	14
**GSE9893**	Chanrion et al., 2008[13]	155	155	155	57
**GSE9195**	Loi et al., 2008[14]	77	77	77	13
**GSE11121**	Schmidt et al., 2008[15]	200	200	200	46
**GSE10510**	Calabrò et al., 2009[28]	139	139	134	96
**GSE16391**	Desmedt et al., 2009[29]	55	55	55	55
**GSE12093**	Zhang et al., 2009[16]	136	136	136	20
**GSE19615**	Li et al., 2010[17]	115	115	115	14
**GSE17907**	Sircoulomb et al., 2010[18]	55	55	39	17
**GSE22219**	Buffa et al., 2011[19]	216	216	216	82
**GSE20711**	Dedeurwaerder et al., 2011[30]	85	85	85	36
**GSE26971**	Filipits et al., 2011[20]	277	277	258	58
**GSE25055**	Hatzis et al., 2011[21]	309	309	309	65
**GSE20685**	Kao et al., 2011[22]	296	296	296	73
**GSE21653**	Sabatier et al., 2011[31]	266	266	252	83
**GSE16987**	Wang et al., 2011[32]	149	149	147	10
**GSE33926**	Kuo et al., 2012[23]	51	51	51	12
**GSE45255**	Nagalla et al., 2013[24]	41	41	41	14
			**Total:**	**5 ,041**	**1 636**

Supplementary Table S4: PLIN1 univariate Cox analysis for MR (Nm; ERm)

Cohort	Reference	*p* value	HR	95% CI	No. patients	No. MR
**Rosetta2002**	Van de Vijver et al., 2002[1]	0.2719	0.89	0.73–1.09	295	101
**GSE2603**	Minn et al., 2005 [2]	0.118	0.73	0.49–1.08	82	27
**GSE1456**	Pawitan et al., 2005 [3]	0.0501	0.74	0.55–1.00	159	40
**GSE2034**	Wang et al., 2005 [4]	0.3381	0.91	0.76–1.10	286	107
**GSE2741**	Weigelt et al., 2005 [5]	0.1989	1.36	0.85–2.17	50	13
**E_TABM_158**	Chin et al., 2006 [6]	0.1831	1.46	0.84–2.57	112	21
**GSE8757**	Chin et al., 2007 [7]	0.031	1.28	1.02–1.60	171	38
**GSE7390**	Desmedt et al., 2007 [8]	0.7977	0.97	0.76–1.24	198	62
**GSE6532**	Loi et al., 2007 [9]	0.0058	0.77	0.64–0.93	393	101
**GSE5327**	Minn et al., 2007 [10]	0.0246	0.42	0.20–0.89	58	11
**GSE7378**	Zhou et al., 2007 [11]	0.2141	0.6	0.27–1.34	54	9
**GSE7849**	Anders et al., 2008 [12]	0.4648	0.78	0.41–1.51	75	14
**GSE9893**	Chanrion et al., 2008 [13]	< 0.0001	0.56	0.45–0.72	155	48
**GSE9195**	Loi et al., 2008 [14]	0.4367	0.79	0.44–1.43	77	10
**GSE11121**	Schmidt et al., 2008 [15]	0.6313	1.07	0.80–1.44	200	46
**GSE12093**	Zhang et al., 2009 [16]	0.0781	0.66	0.42–1.05	136	20
**GSE19615**	Li et al., 2010 [17]	0.2332	0.72	0.43–1.23	115	14
**GSE17907**	Sircoulomb et al., 2010 [18]	0.13	1.63	0.87–3.06	39	17
**GSE22219**	Buffa et al., 2011 [19]	0.2976	0.88	0.70–1.11	216	82
**GSE26971**	Filipits et al., 2011 [20]	0.963	0.99	0.77–1.28	258	58
**GSE25055**	Hatzis et al., 2011 [21]	0.2886	0.87	0.67–1.13	309	65
**GSE20685**	Kao et al., 2011 [22]	0.8471	1.02	0.80–1.31	296	63
**GSE33926**	Kuo et al., 2012 [23]	0.0464	1.52	1.01–2.29	51	12
**GSE45255**	Nagalla et al., 2013 [24]	0.2969	0.75	0.44–1.28	41	14
	Pool	**0.0003**	**0.89**	**0.84–0.95**	**3,826**	**993**

”Pool” consists in merging all cohorts pooled together with data from all studies previously converted to a common scale with a suitable normalisation (median centred [0] and standard deviation normalized to one).

Supplementary Table S5: PLIN1 univariate Cox analysis for AE (Nm; ERm)

Cohort	Reference	*p*-value	HR	95% CI	No. patients	No. AE
**Rosetta2002**	Van de Vijver et al., 2002 [1]	0.1054	0.86	0.72–1.03	295	122
**GSE2603**	Minn et al., 2005 [2]	0.118	0.73	0.49–1.08	82	27
**GSE1456**	Pawitan et al., 2005 [3]	0.3313	0.87	0.66–1.15	159	50
**GSE2034**	Wang et al., 2005 [4]	0.3381	0.91	0.76–1.10	286	107
**GSE2741**	Weigelt et al., 2005 [5]	0.1989	1.36	0.85–2.17	50	13
**GSE3143**	Bild et al., 2006 [25]	0.6482	0.93	0.68–1.27	158	50
**E_TABM_158**	Chin et al., 2006 [6]	0.092	1.39	0.95–2.03	112	42
**GSE4922**	Ivshina et al., 2006 [26]	0.0688	0.83	0.68–1.01	249	89
**GSE8757**	Chin et al., 2007 [7]	0.0956	1.19	0.97–1.47	171	56
**GSE7390**	Desmedt et al., 2007 [8]	0.7242	1.04	0.85–1.27	198	91
**GSE6532**	Loi et al., 2007 [9]	0.029	0.84	0.71–0.98	393	139
**GSE5327**	Minn et al., 2007 [10]	0.0246	0.42	0.20–0.89	58	11
**E_UCON_1**	Naderi et al., 2007 [27]	0.2651	0.86	0.66–1.12	135	65
**GSE7378**	Zhou et al., 2007 [11]	0.2141	0.6	0.27–1.34	54	9
**GSE7849**	Anders et al., 2008 [12]	0.4648	0.78	0.41–1.51	75	14
**GSE9893**	Chanrion et al., 2008 [13]	< 0.0001	0.63	0.50–0.78	155	57
**GSE9195**	Loi et al., 2008 [14]	0.9842	0.99	0.57–1.74	77	13
**GSE11121**	Schmidt et al., 2008 [15]	0.6313	1.07	0.80–1.44	200	46
**GSE10510**	Calabrò et al., 2009 [28]	0.56	0.94	0.76–1.16	134	96
**GSE16391**	Desmedt et al., 2009 [29]	0.9665	1.01	0.78–1.29	55	55
**GSE12093**	Zhang et al., 2009 [16]	0.0781	0.66	0.42–1.05	136	20
**GSE19615**	Li et al., 2010 [17]	0.2332	0.72	0.43–1.23	115	14
**GSE17907**	Sircoulomb et al., 2010 [18]	0.13	1.63	0.87–3.06	39	17
**GSE22219**	Buffa et al., 2011 [19]	0.2976	0.88	0.70–1.11	216	82
**GSE20711**	Dedeurwaerder et al., 2011 [30]	0.4357	1.14	0.82–1.61	85	36
**GSE26971**	Filipits et al., 2011 [20]	0.963	0.99	0.77–1.28	258	58
**GSE25055**	Hatzis et al., 2011 [21]	0.2886	0.87	0.67–1.13	309	65
**GSE20685**	Kao et al., 2011 [22]	0.5655	1.07	0.85–1.34	296	73
**GSE21653**	Sabatier et al., 2011 [31]	0.2943	0.89	0.71–1.11	252	83
**GSE16987**	Wang et al., 2011 [32]	0.0956	0.5	0.22–1.13	147	10
**GSE33926**	Kuo et al., 2012 [23]	0.0464	1.52	1.01–2.29	51	12
**GSE45255**	Nagalla et al., 2013 [24]	0.2969	0.75	0.44–1.28	41	14
	Pool	**0.0006**	**0.92**	**0.87–0.96**	**5,041**	**1636**

Supplementary Table S6: Molecular subtype prognostic analyses for PLIN1 with patients with any event information (metastatic or any relapse, or death) (n = 4,155)

Subtype	Original data				Filtered/Final data									
	No. SSP classified patients				SSP classified patients and AE									
	Sorlie's [33]	Hu's [34]	Parker's [35]	RSSPC	Sorlie's		Hu's		Parker's		RSSPC		RMSPC	
					n	AE	*n*	AE	*n*	AE	*n*	AE	*n*	AE
**Basal-like**	795	1,268	1,144	703	790	254	1,092	366	910	293	699	224	580	193
**HER2-E**	606	502	828	190	601	232	396	154	640	251	190	78	124	49
**Luminal A**	1,503	1,339	1,581	761	1484	383	1,074	204	1,234	254	756	139	324	58
**Luminal B**	637	989	1,068	190	627	247	762	318	786	305	186	94	80	44
**Normal breast-like**	663	808	728	335	653	150	658	159	536	140	332	77	--	--
**Unclassified**	0	0	0	0	0	--	0	--	0	--	--	--	--	--
**Total:**	4,204	4,906	5,349	2,179	4,155	1,266	3,982	1,201	4,106	1,243	2,163	612	1,108	344

AE: any event (any relapse or death); *n*: number of patients; SSP: single sample predictor; MSP: molecular subtype predictor; RMSPC: robust Molecular Subtype Predictors Classification based on patients classified in the same subtype with the six MSPs; RSSPC: robust SSP classification based on patients classified in the same subtype with the three SSPs.

REFERENCES

1. van de Vijver MJ, He YD, van't Veer LJ, Dai H, Hart AA, Voskuil DW, Schreiber GJ, Peterse JL, Roberts C, Marton MJ, Parrish M, Atsma D, Witteveen A, et al. A gene-expression signature as a predictor of survival in breast cancer. N Engl J Med. 2002; 347:1999–2009.
2. Minn AJ, Gupta GP, Siegel PM, Bos PD, Shu W, Giri DD, Viale A, Olshen AB, Gerald WL, Massague J. Genes that mediate breast cancer metastasis to lung. Nature. 2005; 436:518–524.
3. Pawitan Y, Bjohle J, Amler L, Borg AL, Egyhazi S, Hall P, Han X, Holmberg L, Huang F, Klaar S, Liu ET, Miller L, Nordgren H, et al. Gene expression profiling spares early breast cancer patients from adjuvant therapy: derived and validated in two population-based cohorts. Breast Cancer Res. 2005; 7:R953–964.
4. Wang Y, Klijn JG, Zhang Y, Sieuwerts AM, Look MP, Yang F, Talantov D, Timmermans M, Meijer-van Gelder ME, Yu J, Jatkoe T, Berns EM, Atkins D, et al. Gene-expression profiles to predict distant metastasis of lymph-node-negative primary breast cancer. Lancet. 2005; 365:671–679.
5. Weigelt B, Hu Z, He X, Livasy C, Carey LA, Ewend MG, Glas AM, Perou CM, Van't Veer LJ. Molecular portraits and 70-gene prognosis signature are preserved throughout the metastatic process of breast cancer. Cancer Res. 2005; 65:9155–9158.
6. Chin K, DeVries S, Fridlyand J, Spellman PT, Roydasgupta R, Kuo WL, Lapuk A, Neve RM, Qian Z, Ryder T, Chen F, Feiler H, Tokuyasu T, et al. Genomic and transcriptional aberrations linked to breast cancer pathophysiologies. Cancer cell. 2006; 10:529–541.
7. Chin SF, Teschendorff AE, Marioni JC, Wang Y, Barbosa-Morais NL, Thorne NP, Costa JL, Pinder SE, van de Wiel MA, Green AR, Ellis IO, Porter PL, Tavare S, et al. High-resolution aCGH and expression profiling identifies a novel genomic subtype of ER negative breast cancer. Genome Biol. 2007; 8:R215.
8. Desmedt C, Piette F, Loi S, Wang Y, Lallemand F, Haibe-Kains B, Viale G, Delorenzi M, Zhang Y, d'Assignies MS, Bergh J, Lidereau R, Ellis P, et al. Strong time dependence of the 76-gene prognostic signature for node-negative breast cancer patients in the TRANSBIG multicenter independent validation series. Clin Cancer Res. 2007; 13:3207–3214.
9. Loi S, Haibe-Kains B, Desmedt C, Lallemand F, Tutt AM, Gillet C, Ellis P, Harris A, Bergh J, Foekens JA, Klijn JG, Larsimont D, Buyse M, et al. Definition of clinically distinct molecular subtypes in estrogen receptor-positive breast carcinomas through genomic grade. J Clin Oncol. 2007; 25:1239–1246.
10. Minn AJ, Gupta GP, Padua D, Bos P, Nguyen DX, Nuyten D, Kreike B, Zhang Y, Wang Y, Ishwaran H, Foekens JA, van de Vijver M, Massague J. Lung metastasis genes couple breast tumor size and metastatic spread. Proc Natl Acad Sci U S A. 2007; 104:6740–6745.
11. Zhou Y, Yau C, Gray JW, Chew K, Dairkee SH, Moore DH, Eppenberger U, Eppenberger-Castori S, Benz CC. Enhanced NF kappa B and AP-1 transcriptional activity associated with antiestrogen resistant breast cancer. BMC cancer. 2007; 7:59.
12. Anders CK, Acharya CR, Hsu DS, Broadwater G, Garman K, Foekens JA, Zhang Y, Wang Y, Marcom K, Marks JR, Mukherjee S, Nevins JR, Blackwell KL, et al. Age-specific differences in oncogenic pathway deregulation seen in human breast tumors. PloS one. 2008; 3:e1373.
13. Chanrion M, Negre V, Fontaine H, Salvetat N, Bibeau F, Mac Grogan G, Mauriac L, Katsaros D, Molina F, Theillet C, Darbon JM. A gene expression signature that can predict the recurrence of tamoxifen-treated primary breast cancer. Clin Cancer Res. 2008; 14:1744–1752.
14. Loi S, Haibe-Kains B, Desmedt C, Wirapati P, Lallemand F, Tutt AM, Gillet C, Ellis P, Ryder K, Reid JF, Daidone MG, Pierotti MA, Berns EM, et al. Predicting prognosis using

molecular profiling in estrogen receptor-positive breast cancer treated with tamoxifen. BMC genomics. 2008; 9:239.

15. Schmidt M, Bohm D, von Torne C, Steiner E, Puhl A, Pilch H, Lehr HA, Hengstler JG, Kolbl H, Gehrmann M. The humoral immune system has a key prognostic impact in node-negative breast cancer. Cancer Res. 2008; 68:5405–5413.
16. Zhang Y, Sieuwerts AM, McGreevy M, Casey G, Cufer T, Paradiso A, Harbeck N, Span PN, Hicks DG, Crowe J, Tubbs RR, Budd GT, Lyons J, et al. The 76-gene signature defines high-risk patients that benefit from adjuvant tamoxifen therapy. Breast Cancer Res Treat. 2009; 116:303–309.
17. Li Y, Zou L, Li Q, Haibe-Kains B, Tian R, Li Y, Desmedt C, Sotiriou C, Szallasi Z, Iglehart JD, Richardson AL, Wang ZC. Amplification of LAPTM4B and YWHAZ contributes to chemotherapy resistance and recurrence of breast cancer. Nat Med. 2010; 16:214–218.
18. Sircoulomb F, Bekhouche I, Finetti P, Adelaide J, Ben Hamida A, Bonansea J, Raynaud S, Innocenti C, Charafe-Jauffret E, Tarpin C, Ben Ayed F, Viens P, Jacquemier J, et al. Genome profiling of ERBB2-amplified breast cancers. BMC cancer. 2010; 10:539.
19. Buffa FM, Camps C, Winchester L, Snell CE, Gee HE, Sheldon H, Taylor M, Harris AL, Ragoussis J. microRNA-associated progression pathways and potential therapeutic targets identified by integrated mRNA and microRNA expression profiling in breast cancer. Cancer Res. 2011; 71:5635–5645.
20. Filipits M, Rudas M, Jakesz R, Dubsky P, Fitzal F, Singer CF, Dietze O, Greil R, Jelen A, Sevelda P, Freibauer C, Muller V, Janicke F, et al. A new molecular predictor of distant recurrence in ER-positive, HER2-negative breast cancer adds independent information to conventional clinical risk factors. Clin Cancer Res. 2011; 17:6012–6020.
21. Hatzis C, Pusztai L, Valero V, Booser DJ, Esserman L, Lluch A, Vidaurre T, Holmes F, Souchon E, Wang H, Martin M, Cotrina J, Gomez H, et al. A genomic predictor of response and survival following taxane-anthracycline chemotherapy for invasive breast cancer. Jama. 2011; 305:1873–1881.
22. Kao KJ, Chang KM, Hsu HC, Huang AT. Correlation of microarray-based breast cancer molecular subtypes and clinical outcomes: implications for treatment optimization. BMC cancer. 2011; 11:143.
23. Kuo WH, Chang YY, Lai LC, Tsai MH, Hsiao CK, Chang KJ, Chuang EY. Molecular characteristics and metastasis predictor genes of triple-negative breast cancer: a clinical study of triple-negative breast carcinomas. PloS one. 2012; 7:e45831.
24. Nagalla S, Chou JW, Willingham MC, Ruiz J, Vaughn JP, Dubey P, Lash TL, Hamilton-Dutoit SJ, Bergh J, Sotiriou C, Black MA, Miller LD. Interactions between immunity, proliferation and molecular subtype in breast cancer prognosis. Genome Biol. 2013; 14:R34.
25. Bild AH, Yao G, Chang JT, Wang Q, Potti A, Chasse D, Joshi MB, Harpole D, Lancaster JM, Berchuck A, Olson JA Jr, Marks JR, et al. Oncogenic pathway signatures in human cancers as a guide to targeted therapies. Nature. 2006; 439:353–357.
26. Ivshina AV, George J, Senko O, Mow B, Putti TC, Smeds J, Lindahl T, Pawitan Y, Hall P, Nordgren H, Wong JE, Liu ET, Bergh J, et al. Genetic reclassification of histologic grade delineates new clinical subtypes of breast cancer. Cancer Res. 2006; 66:10292–10301.
27. Naderi A, Teschendorff AE, Barbosa-Morais NL, Pinder SE, Green AR, Powe DG, Robertson JF, Aparicio S, Ellis IO, Brenton JD, Caldas C. A gene-expression signature to predict survival in breast cancer across independent data sets. Oncogene. 2007; 26:1507–1516.
28. Calabro A, Beissbarth T, Kuner R, Stojanov M, Benner A, Asslaber M, Ploner F, Zatloukal K, Samonigg H, Poustka A, Sultmann H. Effects of infiltrating lymphocytes and estrogen receptor on gene expression and prognosis in breast cancer. Breast Cancer Res Treat. 2009; 116:69–77.
29. Desmedt C, Giobbie-Hurder A, Neven P, Paridaens R, Christiaens MR, Smeets A, Lallemand F, Haibe-Kains B, Viale G, Gelber RD, Piccart M, Sotiriou C. The Gene expression Grade Index: a potential predictor of relapse for endocrine-treated breast cancer patients in the BIG 1–98 trial. BMC Med Genomics. 2009; 2:40.
30. Dedeurwaerder S, Desmedt C, Calonne E, Singhal SK, Haibe-Kains B, Defrance M, Michiels S, Volkmar M, Deplus R, Luciani J, Lallemand F, Larsimont D, Toussaint J, et al. DNA methylation profiling reveals a predominant immune component in breast cancers. EMBO Mol Med. 2011; 3:726–741.
31. Sabatier R, Finetti P, Cervera N, Lambaudie E, Esterni B, Mamessier E, Tallet A, Chabannon C, Extra JM, Jacquemier J, Viens P, Birnbaum D, Bertucci F. A gene expression signature identifies two prognostic subgroups of basal breast cancer. Breast Cancer Res Treat. 2011; 126:407–420.
32. Wang DY, Done SJ, McCready DR, Boerner S, Kulkarni S, Leong WL. A new gene expression signature, the ClinicoMolecular Triad Classification, may improve prediction and prognostication of breast cancer at the time of diagnosis. Breast Cancer Res. 2011; 13:R92.
33. Sorlie T, Tibshirani R, Parker J, Hastie T, Marron JS, Nobel A, Deng S, Johnsen H, Pesich R, Geisler S, Demeter J, Perou CM, Lonning PE, et al. Repeated observation of breast tumor subtypes in independent gene expression data sets. Proc Natl Acad Sci U S A. 2003; 100:8418–8423.
34. Hu Z, Fan C, Oh DS, Marron JS, He X, Qaqish BF, Livasy C, Carey LA, Reynolds E, Dressler L, Nobel A, Parker J, Ewend MG, et al. The molecular portraits of breast tumors are conserved across microarray platforms. BMC Genomics. 2006; 7:96.
35. Parker JS, Mullins M, Cheang MC, Leung S, Voduc D, Vickery T, Davies S, Fauron C, He X, Hu Z, Quackenbush JF, Stijleman IJ, Palazzo J, et al. Supervised risk predictor of breast cancer based on intrinsic subtypes. J Clin Oncol. 2009; 27:1160–1167.
