# Supplementary material for: Prognostic significance of PLIN1 expression in human breast cancer
Source: Oncotarget. 2016 Jun 23;7(34):54488–502. doi: 10.18632/oncotarget.10239 (PMC5342357; doi:10.18632/oncotarget.10239)
Supplement: Supplementary file 2 [file oncotarget-07-54488-s002.docx]

**Supplementary Table S1:** List of concordant differentially expressed genes

| **S.no** | **Gene Symbol** | **Gene Name** | **Chromosome Location** | **Entrez Gene ID** | **FoldChange** | **log2 Fold Change** | **FDR** | ***p*-value** | **Regulation** |
| --- | --- | --- | --- | --- | --- | --- | --- | --- | --- |
| 1 | KCNJ16 | potassium voltage-gated channel subfamily J member 16 | 17q24.3 | 3773 | 0.052 | −4.27 | 6.47E-17 | 3.21E-21 | down |
| 2 | FIGF | c-fos induced growth factor (vascular endothelial growth factor D) | Xp22.31 | 2277 | 0.044 | −4.50 | 4.69E-14 | 6.00E-18 | down |
| 3 | CA4 | carbonic anhydrase IV | 17q23 | 762 | 0.024 | −5.36 | 4.69E-14 | 6.98E-18 | down |
| 4 | ANGPTL7 | angiopoietin like 7 | 1p36 | 10218 | 0.051 | −4.28 | 6.20E-14 | 1.23E-17 | down |
| 5 | GLYAT | glycine-N-acyltransferase | 11q12.1 | 10249 | 0.031 | −4.98 | 3.85E-13 | 1.15E-16 | down |
| 6 | HEPN1 | hepatocellular carcinoma, down-regulated 1 | 11q24 | 641654 | 0.042 | −4.57 | 2.22E-12 | 7.72E-16 | down |
| 7 | GPD1 | glycerol-3-phosphate dehydrogenase 1 | 12q13.12 | 2819 | 0.053 | −4.21 | 4.73E-11 | 3.43E-14 | down |
| 8 | HSPB7 | heat shock protein family B (small) member 7 | 1p36.13 | 27129 | 0.057 | −4.12 | 2.55E-10 | 2.40E-13 | down |
| 9 | AQP7 | aquaporin 7 | 9p13 | 364 | 0.056 | −4.13 | 5.14E-10 | 6.37E-13 | down |
| 10 | PLIN1 | perilipin 1 | 15q26 | 5346 | 0.054 | −4.18 | 1.05E-09 | 1.78E-12 | down |
| 11 | HEPACAM | hepatic and glial cell adhesion molecule | 11q24.2 | 220296 | 0.051 | −4.27 | 1.50E-09 | 2.60E-12 | down |
| 12 | KCNIP2 | potassium voltage-gated channel interacting protein 2 | 10q24 | 30819 | 0.052 | −4.26 | 1.98E-09 | 3.54E-12 | down |
| 13 | PCK1 | phosphoenolpyruvate carboxykinase 1 | 20q13.31 | 5105 | 0.034 | −4.86 | 5.39E-09 | 1.07E-11 | down |
| 14 | C14orf180 | chromosome 14 open reading frame 180 | 14q32.33 | 400258 | 0.052 | −4.26 | 1.42E-08 | 3.60E-11 | down |
| 15 | TUSC5 | tumor suppressor candidate 5 | 17p13.3 | 286753 | 0.054 | −4.21 | 5.97E-08 | 1.91E-10 | down |
| 16 | PLIN4 | perilipin 4 | 19p13.3 | 729359 | 0.048 | −4.35 | 1.11E-07 | 4.07E-10 | down |
| 17 | LEP | leptin | 7q31.3 | 3952 | 0.024 | −5.33 | 2.54E-07 | 1.17E-09 | down |
| 18 | SLC19A3 | solute carrier family 19 member 3 | 2q37 | 80704 | 0.053 | −4.23 | 3.53E-07 | 1.70E-09 | down |
| 19 | ADIPOQ | adiponectin, C1Q and collagen domain containing | 3q27 | 9370 | 0.057 | −4.11 | 3.65E-07 | 1.78E-09 | down |
| 20 | GPR144 | adhesion G protein-coupled receptor D2 | 9q33.3 | 347088 | 0.026 | −5.25 | 4.71E-07 | 2.45E-09 | down |
| 21 | MMP11 | matrix metallopeptidase 11 | 22q11.23 | 4320 | 39.131 | 5.29 | 8.63E-07 | 5.44E-09 | up |
| 22 | AQP7P3 | aquaporin 7 pseudogene 3 | 9p12 | 441432 | 0.041 | −4.61 | 9.33E-07 | 6.01E-09 | down |
| 23 | SCARA5 | scavenger receptor class A member 5 | 8p21.1 | 286133 | 0.054 | −4.21 | 4.61E-06 | 4.30E-08 | down |
| 24 | ACVR1C | activin A receptor type IC | 2q24.1 | 130399 | 0.042 | −4.55 | 5.38E-06 | 5.33E-08 | down |
| 25 | APOB | apolipoprotein B | 2p24-p23 | 338 | 0.048 | −4.35 | 1.24E-05 | 1.41E-07 | down |
| 26 | MYBL2 | v-myb avian myeloblastosis viral oncogene homolog-like 2 | 20q13.1 | 4605 | 16.483 | 4.04 | 1.54E-05 | 1.80E-07 | up |
| 27 | EPR1 | effector cell peptidase receptor 1 (non-protein coding) | 17q25 | 8475 | 18.010 | 4.10 | 1.90E-05 | 2.38E-07 | up |
| 28 | SCT | secretin | 11p15.5 | 6343 | 21.812 | 4.44 | 2.08E-05 | 2.69E-07 | up |
| 29 | ADH1B | alcohol dehydrogenase 1B (class I), beta polypeptide | 4q23 | 125 | 0.0574 | −4.11 | 3.82E-05 | 5.49E-07 | down |
| 30 | MYOC | myocilin, trabecular meshwork inducible glucocorticoid response | 1q23-q24 | 4653 | 0.0384 | −4.69 | 3.92E-05 | 5.71E-07 | down |
| 31 | PPAPDC1A | phospholipid phosphatase 4 | 10q26.12 | 196051 | 39.123 | 5.29 | 7.26E-05 | 1.19E-06 | up |
| 32 | TIMP4 | TIMP metallopeptidase inhibitor 4 | 3p25 | 7079 | 0.052 | −4.15 | 7.33E-05 | 1.20E-06 | down |
| 33 | LST-3TM12 | solute carrier organic anion transporter family member 1B7 (putative) | 12p12.2 | 338821 | 0.011 | −6.84 | 7.80E-05 | 1.32E-06 | down |
| 34 | CIDEA | cell death-inducing DFFA-like effector a | 18p11.21 | 1149 | 0.061 | −4.15 | 9.51E-05 | 1.70E-06 | down |
| 35 | GJB2 | gap junction protein beta 2 | 13q11-q12 | 2706 | 23.407 | 4.54 | 0.000115 | 2.12E-06 | up |
| 36 | UBE2C | ubiquitin conjugating enzyme E2C | 20q13.12 | 11065 | 16.605 | 4.05 | 0.000129 | 2.45E-06 | up |
| 37 | COL10A1 | collagen type X alpha 1 | 6q21-q22 | 1300 | 40.834 | 5.35 | 0.000223 | 4.62E-06 | up |
| 38 | GRM4 | glutamate receptor, metabotropic 4 | 6p21.3 | 2914 | 61.587 | 5.94 | 0.000412 | 9.93E-06 | up |
| 39 | GLRA3 | glycine receptor alpha 3 | 4q34.1 | 8001 | 0.051 | −4.24 | 0.000452 | 1.12E-05 | down |
| 40 | PITX1 | paired like homeodomain 1 | 5q31.1 | 5307 | 17.132 | 4.09 | 0.000548 | 1.42E-05 | up |
| 41 | PNLIPRP3 | pancreatic lipase related protein 3 | 10q25.3 | 119548 | 0.036 | −4.79 | 0.000559 | 1.45E-05 | down |
| 42 | CST4 | cystatin S | 20p11.21 | 1472 | 87.097 | 6.44 | 0.000658 | 1.75E-05 | up |
| 43 | CSF3 | colony stimulating factor 3 | 17q11.2-q12 | 1440 | 0.041 | −4.58 | 0.000846 | 2.39E-05 | down |
| 44 | TAT | tyrosine aminotransferase | 16q22.1 | 6898 | 0.024 | −5.38 | 0.000982 | 2.86E-05 | down |
| 45 | IBSP | integrin binding sialoprotein | 4q21.1 | 3381 | 152.541 | 7.25 | 0.002425 | 8.31E-05 | up |
| 46 | AADAC | arylacetamide deacetylase | 3q25.1 | 13 | 0.054 | −4.20 | 0.002521 | 8.71E-05 | down |
| 47 | ESM1 | endothelial cell specific molecule 1 | 5q11.2 | 11082 | 16.420 | 4.03 | 0.002536 | 8.79E-05 | up |
| 48 | COL11A1 | collagen type XI alpha 1 | 1p21 | 1301 | 97.554 | 6.61 | 0.002993 | 0.000109 | up |
| 49 | CST2 | cystatin SA | 20p11.21 | 1470 | 38.585 | 5.27 | 0.003794 | 0.000145 | up |
| 50 | ARHGAP36 | Rho GTPase activating protein 36 | Xq26.1 | 158763 | 0.024 | −5.33 | 0.004693 | 0.000188 | down |
| 51 | IGFL3 | IGF like family member 3 | 19q13.32 | 388555 | 30.200 | 4.91 | 0.005288 | 0.000217 | up |
| 52 | NPW | neuropeptide W | 16p13.3 | 283869 | 62.046 | 5.95 | 0.005319 | 0.000219 | up |
| 53 | LHCGR | luteinizing hormone/choriogonadotropin receptor | 2p21 | 3973 | 0.045 | −4.45 | 0.00539 | 0.000222 | down |
| 54 | CST1 | cystatin SN | 20p11.21 | 1469 | 36.553 | 5.19 | 0.005703 | 0.000238 | up |
| 55 | SULT1C3 | sulfotransferase family 1C member 3 | 2q12.3 | 442038 | 0.006 | −7.22 | 0.006313 | 0.000271 | down |
| 56 | EPYC | epiphycan | 12q21 | 1833 | 151.484 | 7.24 | 0.006473 | 0.000278 | up |
| 57 | NKAIN1 | Na+/K+ transporting ATPase interacting 1 | 1p35.2 | 79570 | 19.535 | 4.28 | 0.008947 | 0.000411 | up |
